# Triplet-pore structure of a highly divergent TOM complex of hydrogenosomes in *Trichomonas vaginalis*

**DOI:** 10.1371/journal.pbio.3000098

**Published:** 2019-01-04

**Authors:** Abhijith Makki, Petr Rada, Vojtěch Žárský, Sami Kereïche, Lubomír Kováčik, Marian Novotný, Tobias Jores, Doron Rapaport, Jan Tachezy

**Affiliations:** 1 Department of Parasitology, Faculty of Science, Charles University, BIOCEV, Prague, Czech Republic; 2 Institute of Biology and Medical Genetics, First Faculty of Medicine, Charles University, Prague, Czech Republic; 3 Department of Cell Biology, Faculty of Science, Charles University, Prague, Czech Republic; 4 Interfaculty Institute of Biochemistry, University of Tübingen, Tübingen, Germany; Universitat Bern, SWITZERLAND

## Abstract

Mitochondria originated from proteobacterial endosymbionts, and their transition to organelles was tightly linked to establishment of the protein import pathways. The initial import of most proteins is mediated by the translocase of the outer membrane (TOM). Although TOM is common to all forms of mitochondria, an unexpected diversity of subunits between eukaryotic lineages has been predicted. However, experimental knowledge is limited to a few organisms, and so far, it remains unsettled whether the triplet-pore or the twin-pore structure is the generic form of TOM complex. Here, we analysed the TOM complex in hydrogenosomes, a metabolically specialised anaerobic form of mitochondria found in the excavate *Trichomonas vaginalis*. We demonstrate that the highly divergent β-barrel *T*. *vaginalis* TOM (TvTom)40-2 forms a translocation channel to conduct hydrogenosomal protein import. TvTom40-2 is present in high molecular weight complexes, and their analysis revealed the presence of four tail-anchored (TA) proteins. Two of them, Tom36 and Tom46, with heat shock protein (Hsp)20 and tetratricopeptide repeat (TPR) domains, can bind hydrogenosomal preproteins and most likely function as receptors. A third subunit, Tom22-like protein, has a short *cis* domain and a conserved Tom22 transmembrane segment but lacks a *trans* domain. The fourth protein, hydrogenosomal outer membrane protein 19 (Homp19) has no known homology. Furthermore, our data indicate that TvTOM is associated with sorting and assembly machinery (Sam)50 that is involved in β-barrel assembly. Visualisation of TvTOM by electron microscopy revealed that it forms three pores and has an unconventional skull-like shape. Although TvTOM seems to lack Tom7, our phylogenetic profiling predicted Tom7 in free-living excavates. Collectively, our results suggest that the triplet-pore TOM complex, composed of three conserved subunits, was present in the last common eukaryotic ancestor (LECA), while receptors responsible for substrate binding evolved independently in different eukaryotic lineages.

## Abstract 2

The highly divergent outer membrane translocase (TOM) from the Trichomonas hydrogenosome (an organelle related to mitochondria) is composed of conserved core and lineage-specific subunits, and has an unconventional skull-like triplet-pore structure.

## Author summary

Mitochondria carry out many vital functions in the eukaryotic cells, from energy metabolism to programmed cell death. These organelles descended from bacterial endosymbionts, and during their evolution, the cell established a mechanism to transport nuclear-encoded proteins into mitochondria. Embedded in the mitochondrial outer membrane is a molecular machine, known as the translocase of the outer membrane (TOM) complex, that plays a key role in protein import and biogenesis of the organelle. Here, we provide evidence that the TOM complex of hydrogenosomes, a metabolically specialised anaerobic form of mitochondria in *Trichomonas vaginalis*, is composed of highly divergent core subunits and lineage-specific peripheral subunits. Despite the evolutionary distance, the *T*. *vaginalis* TOM (TvTOM) complex has a conserved triplet-pore structure but with a unique skull-like shape suggesting that the TOM in the early mitochondrion could have formed three pores. Our results contribute to a better understanding of the evolution and adaptation of protein import machinery in anaerobic forms of mitochondria.

## Introduction

Mitochondria originated from proteobacterial endosymbionts [1], and over time, massive endosymbiotic gene transfer to the host nucleus or gene deletion forged the development of a mechanism for retargeting of nuclear-encoded proteins to the evolving organelle [2]. To cross the double membrane of the mitochondrion, the proteins had to pass through the translocase of the outer (TOM) and inner (TIM) membranes. It has been inferred that most modules of the import machinery were created de novo and the ancient TOM complex comprised at least three components, the β-barrel translocation channel-forming Tom40 and two tail-anchored (TA) proteins, Tom22 and Tom7 [3,4].

The TOM complex in yeast consists of Tom40 and six α-helical proteins: two that are anchored to the outer mitochondrial membrane (OMM) by an N-terminal transmembrane domain (TMD; Tom20 and Tom70) and four that are anchored by a C-terminal TMD (Tom22, Tom5, Tom6, and Tom7). Tom20 and Tom70, both carrying tetratricopeptide repeat (TPR) domains, serve as primary receptors recognising proteins with N-terminal targeting sequence (NTS) and internal-targeting sequences (ITSs), respectively [5,6]. A prominent feature of the TOM complex is the variation in receptors across different eukaryotic lineages. A signal-anchored Tom20 is present in animals and fungi, whereas plant Tom20 evolved independently with a C-terminal anchor [7]. Lineage-specific Tom20 and Tom60 without any TMD are present in amoebozoans [8,9]. Tom20 and Tom70 are essentially absent in the eukaryotic supergroup Excavata [10–12]. In the excavate *Trypanosoma brucei*, the TOM complex (named the archaic translocase of the outer membrane [ATOM]) has only two orthologues, a highly divergent Tom40 (ATOM40) and a Tom22-like protein (ATOM14) [11,13]. Instead of Tom70 and Tom20, two unique receptors were identified, a TA protein ATOM69 and a signal-anchored ATOM46 [11].

Structural studies of the contemporary TOM complex are exclusively based on fungi, *Saccharomyces cerevisiae* and *Neurospora crassa* [14,15]. The yeast TOM complex is highly dynamic, with the mature trimeric complex formed by three pores, alternately switching with a dimeric form containing two pores, which serves as a platform for the integration of a new Tom40 into the complex [16]. The assembly of the Tom40 precursor in the OMM is mediated by the sorting and assembly machinery (SAM) that consists of a central β-barrel subunit Sam50 and two peripheral subunits Sam35 and Sam37 in yeast. To promote β-barrel biogenesis, TOM and SAM form a transient supercomplex [17,18]. The dimeric and trimeric TOM structures are stabilised by the highly conserved TMD of Tom22 [19]. This specific function of Tom22 and its conservation in most eukaryotes led to speculation that the ancient TOM complex may have been a trimeric form [12]. However, this concept remains unsettled as it has not been clarified whether *N*. *crassa* TOM complex forms a three-pore or a two-pore structure [15,20], and so far, the information on TOM structure from other organisms is unavailable. Thus, to understand what subunits contributed to the formation of the earliest translocases and to reconstruct the evolutionary steps, it is important to study the composition and the structure of the translocases in organisms harbouring different variants of mitochondria as well as in organisms from different eukaryotic supergroups. Highly reduced mitochondria known as hydrogenosomes and mitosomes are found in certain organisms adapted to an anaerobic lifestyle [21] with simplified import machinery. The most studied hydrogenosomes are those found in the Parabasalia group of excavates, which includes the human parasite *Trichomonas vaginalis*. *T*. *vaginalis* hydrogenosomes have lost the tricarboxylic acid (TCA) cycle, and the oxidative phosphorylation has been replaced by substrate-level ATP synthesis, with the concomitant production of hydrogen [22]. Hydrogenosomes have lost the organellar genome entirely [23], and consequently, all hydrogenosomal proteins are imported from the cytosol. Like mitochondria, the import of proteins into hydrogenosomes is dependent on the hydrogenosomal NTS [24]. However, some matrix proteins are imported into hydrogenosomes independent of an NTS, and therefore the NTS-independent route was proposed to represent an ancestral mode of protein import [25,26]. Previous proteomic analysis of *T*. *vaginalis* hydrogenosomes revealed the presence of several β-barrel proteins of the mitochondrial porin 3 superfamily that were designated as putative Tom40. However, the protein sequences were highly divergent from known homologues, making it difficult to unequivocally distinguish between Tom40 and voltage-dependent anion channel (VDAC) [10]. Other hydrogenosomal β-barrel proteins include Sam50 and paralogues of two proteins of unknown function, hydrogenosomal membrane protein 35 (Hmp35) and Hmp36 [10,27]. Neither genomic nor proteomic analyses indicated the presence of other TOM components [10,28]. Hydrogenosomes also lack Tim50 and its regulatory subunit Tim21 that links the TOM complex with TIM in the intermembrane space (IMS) [10,28,29]. Furthermore, five paralogues of the Tim17/22/23 family that constitute the TIM channel have been detected. However, limited similarity of these hydrogenosomal proteins to Tim17, Tim22, and Tim23 subfamilies prevented determining whether they form a single multifunctional channel or distinct TIM23 and TIM22 channels for the import of matrix and inner membrane proteins, respectively [10]. Thus, structure and function of the hydrogenosomal protein import machineries remains elusive.

In the present study, we focus on the *T*. *vaginalis* TOM complex (TvTOM) and demonstrate that this highly divergent translocase mediates protein import into hydrogenosomes. Despite remarkable divergence in both primary structure and evolutionary distance, electron microscopy revealed some structural similarity between TvTOM and yeast three-pore TOM complex. However, the presence of an extra density provides a unique skull-like shape to TvTOM. Mass spectrometry (MS) of TvTOM and bioinformatic analysis identified two conserved and three lineage-specific TOM subunits, including two receptors, and revealed an association of TvTOM with Sam50. Although we did not identify Tom7 in TvTOM, our phylogenetic profiling predicted Tom7 in free-living representatives of Excavata. We propose that Tom40, Tom22, and probably Tom7 were present in the last common eukaryotic ancestor (LECA) and constituted a triplet-pore TOM complex, whereas the receptor subunits evolved independently in different eukaryotic lineages.

## Results

### Bioinformatic analyses of Tom40-like proteins

Seven Tom40-like proteins, named TvTom40-1 to TvTom40-7, identified in the hydrogenosomal proteome [10] displayed remarkably low sequence identity with fungal Tom40 sequences (e.g., 10%–14% identity compared with *N*. *crassa*). All TvTom40 proteins carry a conserved β-motif, PxGxxHxH (P = polar; G = glycine; H = hydrophobic; x = any amino acid) in the last β-strand similar to Tom40s and VDACs of other eukaryotes except TvTom40-3, where the last hydrophobic amino acid has been replaced by a polar hydroxylic residue, serine (S1 Fig). Bioinformatic analyses for all the seven proteins using HHpred tool identified TvTom40-2 (TVAG_332970) as the closest homologue to Tom40 (S1 and S2 Tables). Next, we built a local Tom40 hidden Markov model (HMM), based on 24 well-annotated Tom40 sequences (S1 Data) that was employed to scan the *T*. *vaginalis* proteome with HMMER jackhmmer tool, and again, TvTom40-2 was identified as the best Tom40 candidate.

**Fig 1:**
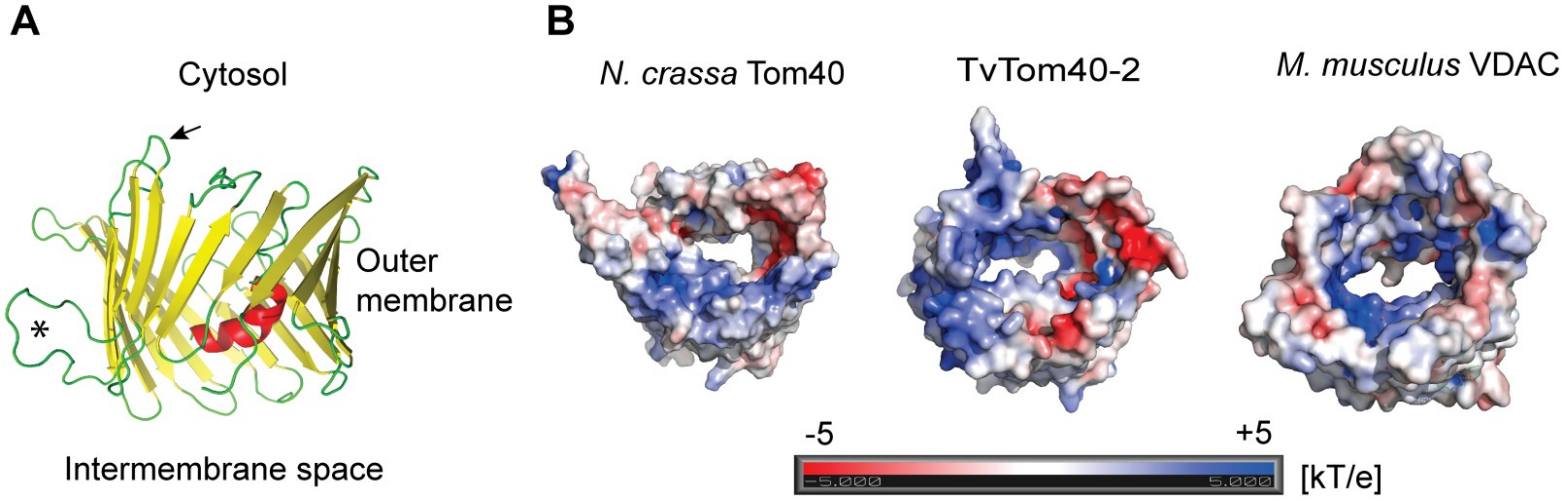
Homology model of TvTom40-2. (A) Model of TvTom40-2 was built using the *N*. *crassa* Tom40 structure (PDB ID 5o8o) as a template. The asterisk shows the extra loop between β-strands five and six, and the arrow shows the loop between β-strands four and five. (B) Comparison of 3D structures of *N*. *crassa* Tom40 (5o8o), TvTom40-2, and *Mus musculus* VDAC (3emn). Mouse VDAC is almost uniformly positively charged inside the barrel to bind negatively charged small molecules (ATP), while TvTom40-2 and *N*. *crassa* Tom40 share both positively and negatively charged patches inside the barrel. The scale of the electrostatic potential ranges from −5 to +5 kT/e. 3D, three-dimensional; PDB, Protein Data Bank; TOM, translocase of the outer membrane; TvTom, *T*. *vaginalis* TOM; VDAC, voltage-dependent anion channel.

A homology model of TvTom40-2 was constructed based on the *N*. *crassa* Tom40 template. TvTom40-2 forms a typical 19-strand β-barrel structure, but with only one N-terminal helix instead of two helices observed in Tom40 of other eukaryotes. Furthermore, TvTom40-2 contains a unique loop between β-strands five and six that is positively charged (Fig 1A). Most of the positions responsible for the interactions with other TOM proteins in yeast [16] are not conserved in TvTom40-2 (S2 Fig). A comparison of the electrostatic potential revealed that TvTom40-2 and *N*. *crassa* Tom40 share both positively and negatively charged patches inside the barrel, whereas mouse VDAC is almost uniformly positively charged (Fig 1B). Hence, based on homology searches and modeling, TvTom40-2 was chosen for further experimental studies.

### TvTom40-2 forms a high molecular weight complex in the hydrogenosomal outer membrane

**Fig 2:**
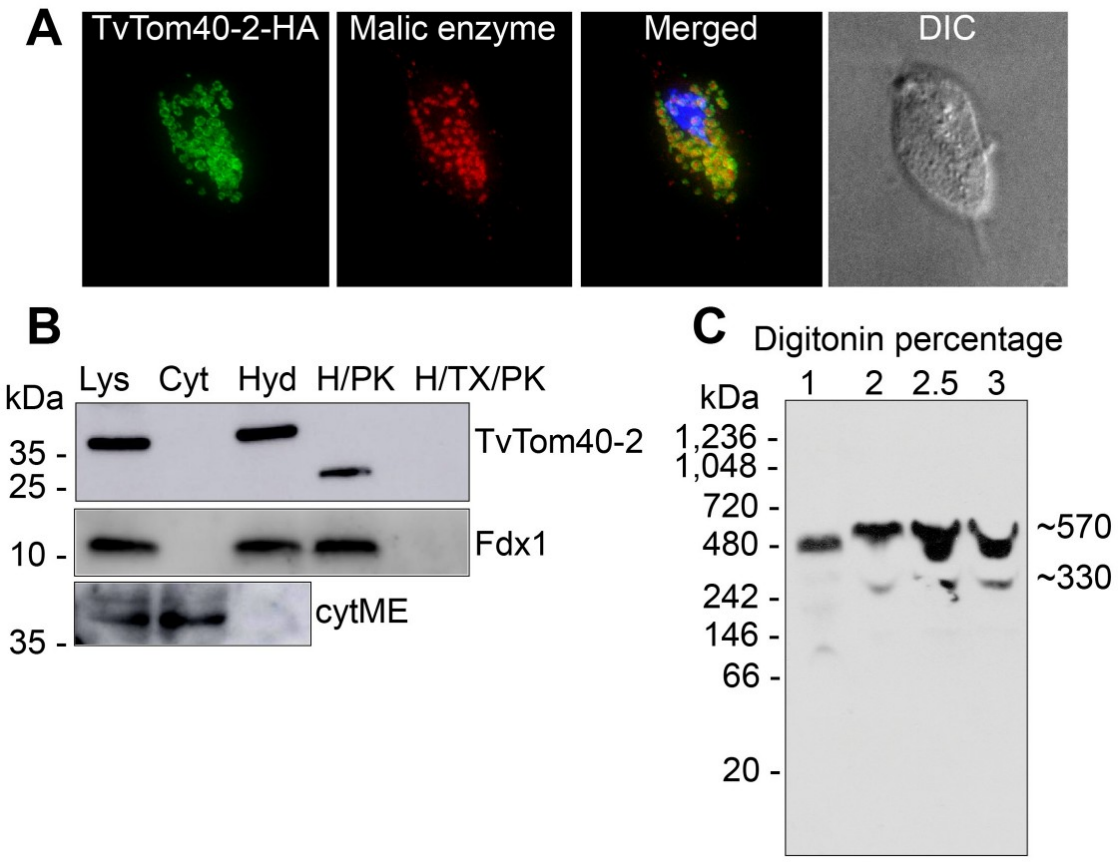
Localisation of TvTom40-2 in the hydrogenosomal outer membrane. (A) HA-tagged TvTom40-2 and malic enzyme (hydrogenosomal matrix protein) were visualised using mouse α-HA (green) and rabbit α-malic enzyme (red) antibodies, respectively. The nucleus was stained with DAPI (blue). (B) Localisation and topology of TvTom40-2 in *T*. *vaginalis* subcellular fractions. Immunoblot analysis of the whole cell lysate, cytoplasm, hydrogenosomes, hydrogenosomes treated with proteinase K, and hydrogenosomes treated with proteinase K in the presence of Triton X-100 using antibodies against HA, Fdx1 (hydrogenosomal matrix protein), and cytosolic malic enzyme. (C) BN-PAGE immunoblots of digitonin-lysed hydrogenosomal extract from the strain expressing HA-tagged TvTom40-2. The samples were probed with α-HA antibody. BN-PAGE, blue native PAGE; Cyt, cytoplasm; cytME, cytoplasmic malic enzyme; DIC, differential interference contrast; Fdx, ferredoxin; H/PK, hydrogenosomes treated with proteinase K; H/TX/PK, hydrogenosomes treated with proteinase K in the presence of Triton X-100; HA, human influenza hemagglutinin; Hyd, hydrogenosomes; Lys, lysate; TOM, translocase of the outer membrane; TvTom, *T*. *vaginalis* TOM.

To verify the cellular localisation of TvTom40-2, a strain expressing C-terminally human influenza hemagglutinin (HA)-tagged TvTom40-2 was prepared. Immunofluorescence microscopy visualised TvTom40-2 as a ring, staining the membrane of hydrogenosomes. Malic enzyme, a hydrogenosomal marker enzyme, stained the organellar matrix (Fig 2A). Cell fractionation and immunoblotting revealed the presence of TvTom40-2 exclusively in the hydrogenosomal fraction (Fig 2B). Treatment of hydrogenosomes carrying HA-tagged TvTom40-2 with proteinase K resulted in a shift of the molecular weight from 37 kDa to 28 kDa, indicating that the protein was likely cleaved within the loop between the fourth and fifth β-strands that is oriented towards the cytosol (Figs 2B and 1A). Then, the isolated hydrogenosomes were solubilised with varying concentrations of digitonin (1%–3%), and the samples were subjected to blue native-PAGE (BN-PAGE). TvTom40-2 was observed to be present in two high molecular weight complexes of 570 kDa and 330 kDa (Fig 2C). These experiments demonstrate that TvTom40-2 is present in a high molecular weight complex embedded in the hydrogenosomal outer membrane.

### TvTom40-2 was inserted into the OMM in *S*. *cerevisiae*

**Fig 3:**
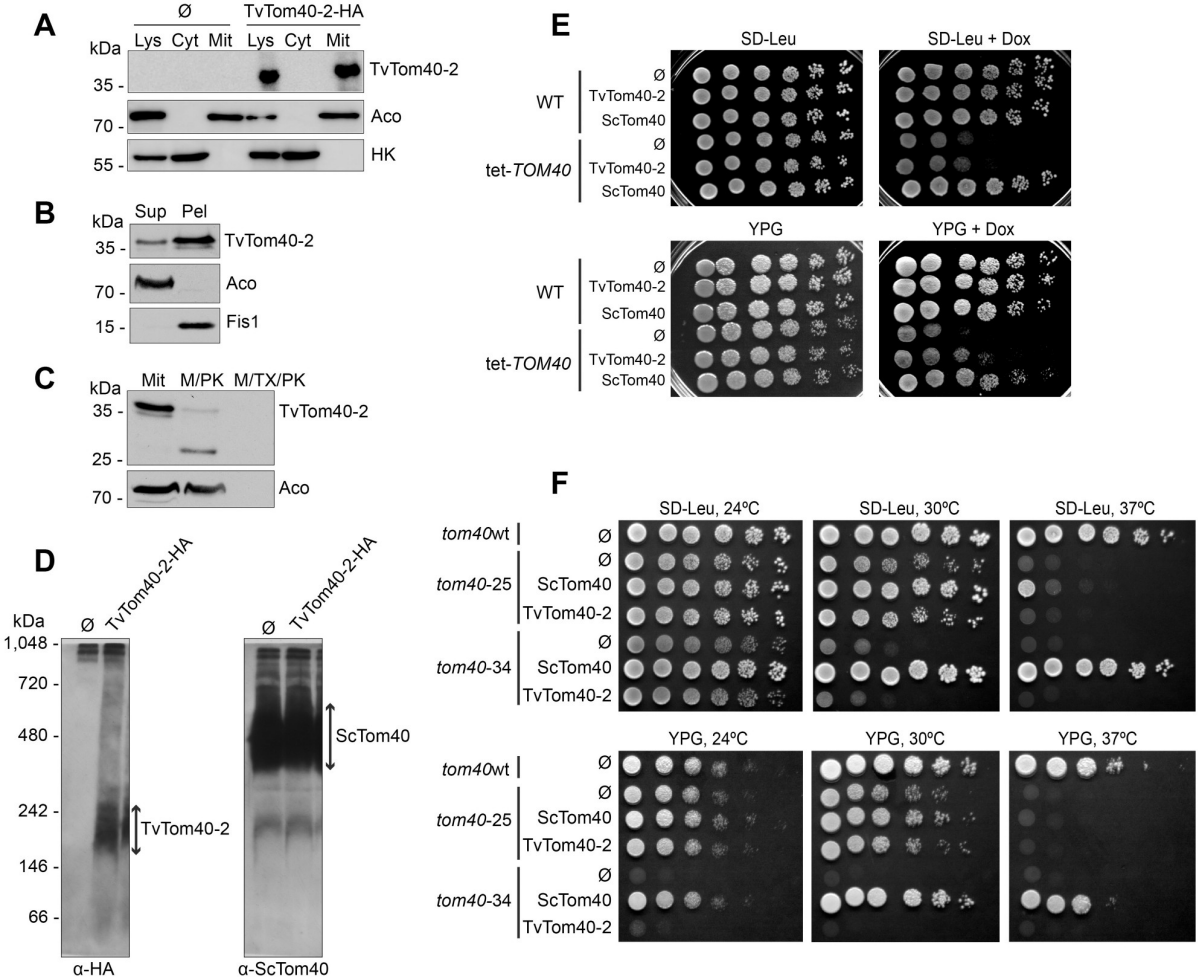
TvTom40-2 was assembled in the mitochondrial outer membrane in *S*. *cerevisiae*, and it could partially rescue the growth phenotype of *TOM40* mutants. (A) Whole cell lysate and fractions corresponding to cytoplasm and mitochondria were obtained from the WT strain transformed with an empty plasmid (Ø) or a plasmid encoding HA-tagged TvTom40-2. Proteins were analysed by SDS-PAGE and immunodecorated with antibodies against HA, aconitase (mitochondrial matrix protein) and hexokinase (cytosolic protein). (B) The mitochondrial fraction of cells expressing HA-tagged TvTom40-2 were subjected to alkaline extraction. Samples corresponding to supernatant and pellet fractions were analysed by western blotting using antibodies against HA, Aco, and Fis1. (C) Mitochondria as in panel B were treated with proteinase K or with proteinase K in the presence of Triton X-100. Further analysis was as in panel A. (D) Mitochondria were isolated from the strains described in panel A and solubilised in digitonin-containing buffer. Samples were analysed by BN-PAGE and immunodecorated with the indicated antibodies. (E) WT and tet-*TOM40* cells transformed with empty plasmid (Ø) or with plasmid encoding either TvTom40-2 or ScTom40 were grown to an OD_600_ of 1.0 and spotted in a 1:5 dilution series on synthetic glucose-containing medium lacking Leucine, SD-Leu supplemented with Dox, rich glycerol-containing medium (YPG), or YPG supplemented with Dox. The plates were then incubated at 30 °C for 2 to 3 days. (F) WT strain transformed with empty plasmid (Ø), or *tom40*-25 and *tom40*-34 strains transformed with empty plasmid (Ø) or with a plasmid encoding either TvTom40-2 or ScTom40, were grown to an OD_600_ of 1.0 and spotted in a 1:5 dilution series on SD-Leu or YPG. The plates were then incubated at 24 °C, 30 °C, or 37 °C for 2 to 4 days. Aco, aconitase; BN-PAGE, blue native PAGE; Cyt, cytoplasm; Dox, doxycycline; Fis1, mitochondrial fission 1; HA, human influenza hemagglutinin; HK, hexokinase; Lys, lysate; M/PK, mitochondria treated with proteinase K; M/TX/PK, mitochondria treated with proteinase K in the presence of Triton X-100; Mit, mitochondria; OD, optical density; Pel, pellet; SD-Leu, synthetic drop-out medium without leucine; SDS-PAGE, sodium dodecyl sulphate-PAGE; Sup, supernatant; TOM, translocase of the outer membrane; TvTom, *T*. *vaginalis* TOM; WT, wild-type; YPG, yeast extract-peptone-glycerol.

The striking divergence of hydrogenosomal TvTom40-2 from Tom40 orthologues prompted us to test whether biogenesis of TvTom40-2 is specific to the hydrogenosomal machinery or whether, despite the variance in the sequence, it could be integrated into the OMM of distant eukaryotes from Opisthokonta lineage. We expressed TvTom40-2 with a C-terminal HA tag in *S*. *cerevisiae*. TvTom40-2 appeared in the mitochondrial fraction together with the mitochondrial marker, aconitase (Fig 3A). Alkaline extraction showed that most of the TvTom40-2 was present, similar to the OMM protein, mitochondrial fission 1 (Fis1), in the membrane fraction (Fig 3B). Finally, treatment of isolated mitochondria with proteinase K resulted in the formation of a proteolytic fragment of TvTom40-2 that resembled the one observed with isolated hydrogenosomes (Fig 3C). As expected, this fragment was completely degraded upon solubilisation of the organelles with the detergent. Collectively, these observations indicate that TvTom40-2 is localised in the OMM in yeast. In addition, to check whether TvTom40-2 could form an oligomeric complex in yeast mitochondria, we performed BN-PAGE. TvTom40-2 migrated in a 230 kDa complex, while ScTom40 migrated in a 480 kDa complex (Fig 3D). This suggests that TvTom40-2 can form in yeast mitochondria a high molecular weight complex, although of smaller size than that in hydrogenosomes.

### TvTom40-2 partially suppresses the growth phenotype of yeast *TOM40* mutants

Because TvTom40-2 was integrated into the OMM of yeast, we wanted to test whether it could functionally replace ScTom40. First, we prepared a yeast mutant, tet-*TOM40*, such that the *TOM40* promoter was replaced by a tetracycline promoter via homologous recombination, which would deplete ScTom40 in the presence of doxycycline (Dox). As expected, the addition of Dox to the growth medium resulted in a growth retardation of the tet-*TOM40* mutant. When TvTom40-2 was overexpressed, it could not rescue the growth defect of the tet-*TOM40* strain on fermentable medium (synthetic drop-out medium without leucine, SD-Leu) but could do so on nonfermentable medium (yeast extract-peptone-glycerol [YPG]) (Fig 3E). To substantiate this observation, we performed functional complementation studies using two yeast strains harbouring temperature-sensitive alleles of *TOM40*—*tom40*-25 and *tom40*-34. When grown at 30 °C, the overexpression of TvTom40-2 partially restored the growth phenotype of the *tom40*-25 strain both on fermentable and nonfermentable media (Fig 3F). Such an effect was not observed in the same strain grown at elevated temperature (37 °C, Fig 3F). The growth of *tom40*-34 was not restored even at lower temperatures (Fig 3F). Thus, it seems that TvTom40-2 can only partially replace yeast Tom40 function.

### Identification of the TvTOM components

**Fig 4:**
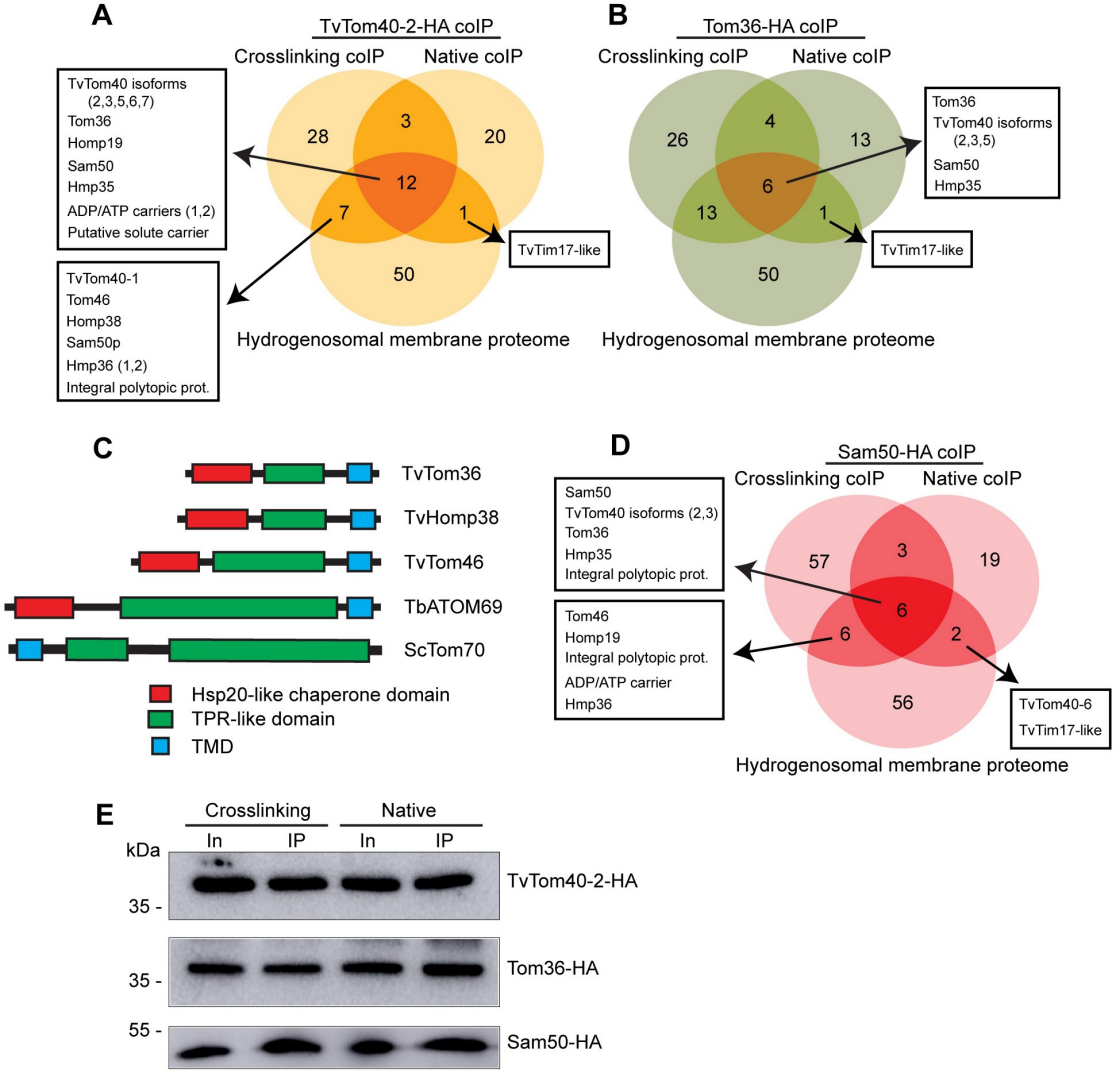
Identification of the components of the TvTOM complex. (A, B) Venn diagrams depicting the intersection between the hydrogenosomal membrane proteome and the proteins identified by LFQ-MS that were enriched in TvTom40-2-HA and Tom36-HA coIPs (under both crosslinking and native conditions), respectively. (C) Scheme of predicted domain architecture of Tom36, Homp38, and Tom46 in comparison with TbATOM69 and ScTom70. Hsp20-like chaperone domain, TPR-like domain, and TMD are represented by blue, green, and red, respectively. (D) Venn diagram depicting the intersection between the hydrogenosomal membrane proteome and the proteins identified by LFQ-MS that were enriched in Sam50-HA coIPs under both crosslinking and native conditions. (E) Immunoblots for the digitonin-lysed extract of hydrogenosomes (Input; 5%) and the IP eluates (2.5%) from TvTom40-2-HA, Tom36-HA, and Sam50-HA coIPs under crosslinking and native conditions decorated with α-HA antibody. ATOM, archaic translocase of the outer membrane; coIP, co-immunoprecipitation; HA, human influenza hemagglutinin; Homp, hydrogenosomal outer membrane protein; Hsp20, heat shock protein 20; In, Input; LFQ-MS, label-free quantitative mass spectrometry; Sam, sorting and assembly machinery; TMD, transmembrane domain; TOM, translocase of the outer membrane; TPR, tetratricopeptide repeat; TvTOM, *T*. *vaginalis* TOM.

To identify interaction partners for TvTom40-2, we performed co-immunoprecipitations (coIPs) of HA-tagged TvTom40-2 under crosslinking and native conditions, and the eluted proteins were analysed using label-free quantitative MS (LFQ-MS). CoIPs using anti-HA antibody were performed with hydrogenosomes isolated from both the strain expressing HA-tagged TvTom40-2 and the wild-type (WT) strain, used as a negative control. The analysis revealed that 50 and 36 proteins were enriched with HA-tagged TvTom40-2 under crosslinking and native conditions, respectively (S2 Data). As TOM proteins are embedded in the hydrogenosomal outer membrane, we searched for proteins with TMDs in the data sets using TMHMM and found 19 and 13 proteins for crosslinking and native coIPs, respectively. The intersection between the two data sets and the hydrogenosomal membrane proteome [10] contained five TvTom40 isoforms, two TA proteins named Tom36 and hydrogenosomal outer membrane protein 19 (Homp19), two Sam50 paralogues, and Hmp35 (S2 Data and Fig 4A). In addition, the intersection between the coIP data set under crosslinking conditions and the membrane proteome contained two more TA proteins named Tom46 and Homp38. Based on our previous results [10], we selected Tom36 for the reciprocal coIPs.

Proteins enriched in the HA-tagged Tom36 coIPs under crosslinking conditions included three isoforms of TvTom40, Sam50, Hmp35, Homp38, Tom46, and Homp19, whereas under native conditions, three isoforms of TvTom40, Sam50, and Hmp35 were enriched (S2 Data and Fig 4B). Altogether, the coIP and MS data indicated four TA candidate proteins, Homp19, Tom36, Homp38, and Tom46. InterProScan [30] predicted that Tom36, Homp38, and Tom46 would carry an N-terminal heat shock protein (Hsp)20-like chaperone domain, three TPR-like domains, and a C-terminal TMD. This domain architecture resembles the recently reported ATOM69 in *T*. *brucei* [11] (Fig 4C). Indeed, HHpred searches using Tom36 and Homp38 as queries against the *T*. *brucei* proteome revealed ATOM69 as the first hit, with e-values of 4.9 × 10^−17^ and 2.3 × 10^−11^, respectively. HHpred searches with Tom46 recognised various proteins with TPR domains, whereas no significant homology was observed for Homp19.

The coIP-MS data did not identify homologues of either Tom22 or Tom7. Thus, we used HMM to search for Tom22 and Tom7 sequences in the *T*. *vaginalis* protein database. The searches for Tom22 identified a small protein with a predicted molecular weight of 6.4 kDa, containing a C-terminal TMD. It has a conserved Tom22 motif, including a tryptophan residue at the second position, followed by a few hydroxylated residues, with a serine at the +4 position and an invariant proline residue in the TMD; hence, we named it Tom22-like protein (TVAG_076160) (S3 Fig). In comparison to the fungal Tom22, Tom22-like protein is substantially shorter, similar to Tom22-like proteins in plants, apicomplexans, and kinetoplastids [4,31,32]. However, unlike Tom22, Tom22-like protein lacks a C-terminal IMS domain (S3 Fig). Searches for Tom7 in the *T*. *vaginalis* protein database did not identify a convincing orthologue.

Interestingly, Sam50 that only transiently associates with TOM in yeast [17] was copurified when both TvTom40-2 and Tom36 were pulled down both under crosslinking and native conditions, which may suggest a more stable association between TvTOM and Sam50. Therefore, we performed reciprocal coIPs using a strain expressing HA-tagged Sam50. LFQ-MS analysis revealed a similar spectrum of proteins as observed in the previous experiments that supports TvTOM-Sam50 association (S2 Data and Fig 4D). The presence of HA-tagged proteins in the eluates from TvTom40-2-HA, Tom36-HA, and Sam50-HA crosslinking and native coIPs were verified via immunoblotting (Fig 4E).

### Hydrogenosomal localisation and the topology of TA proteins

**Fig 5:**
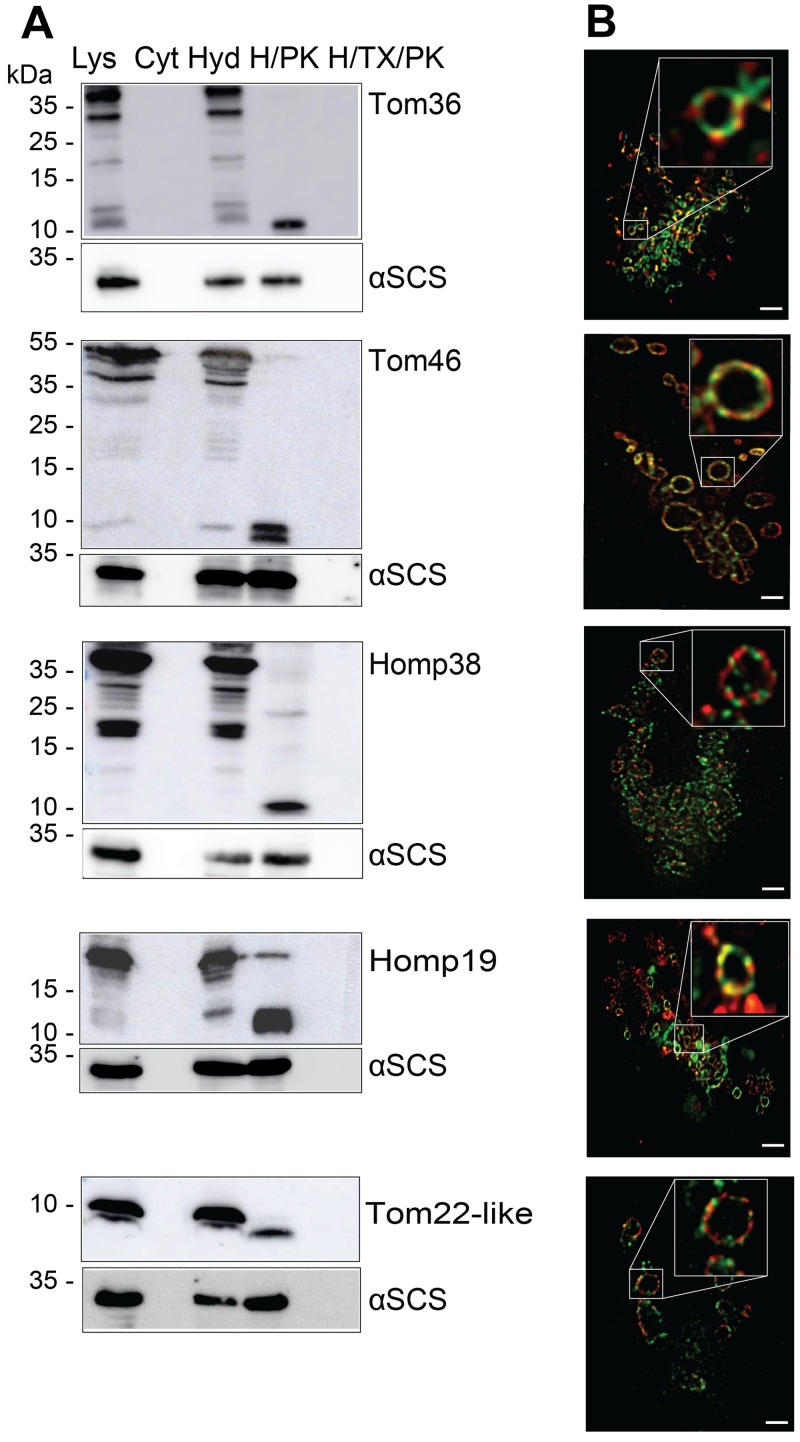
Localisation and topology of the TA proteins. (A) Immunoblot analysis of TA proteins in *T*. *vaginalis* subcellular fractions using α-V5 and α-αSCS (hydrogenosomal matrix protein) antibodies. Total cell lysates, cytoplasm, hydrogenosomes, hydrogenosomes treated with either proteinase K, or hydrogenosomes treated with proteinase K and Triton X-100 isolated from the strains expressing V5-tagged Tom36, Tom46, Homp38, Homp19, and Tom22-like protein. (B) Double transfectants expressing HA-tagged TvTom40-2 along with one of the V5-tagged proteins, Tom36, Tom46, Homp38, Homp19 or Tom22-like protein were visualised using mouse α-HA/α-mouse Abberior STAR 580 (green) and rabbit α-V5/α-rabbit Abberior STAR 635p (red) antibodies. Scale bar, 1 μm. αSCS, α-subunit of succinyl CoA synthetase; CoA, coenzyme A; Cyt, cytoplasm; H/PK, hydrogenosomes treated with proteinase K; H/TX/PK, hydrogenosomes treated with proteinase K in the presence of Triton X-100; HA, human influenza hemagglutinin; Homp, hydrogenosomal outer membrane protein; Hyd, hydrogenosomes; Lys, lysate; TA, tail-anchored; TOM, translocase of the outer membrane; TvTom, *T*. *vaginalis* TOM.

To verify the localisation and topology of identified TA proteins, we prepared double transfectants that expressed TvTom40-2-HA together with one of the candidate proteins, all of which were C-terminally tagged with V5. In all cases, the TA protein was present in the hydrogenosomal fraction (Fig 5A). Treatment of isolated hydrogenosomes with proteinase K showed the presence of a truncated fragment that was protected from externally added proteinase K (Fig 5A). Next, we visualised V5-tagged candidate proteins, together with HA-tagged TvTom40-2, in the double transfectants using Stimulated Emission Depletion (STED) microscopy. All five candidates exhibited a ring-like pattern in the hydrogenosomal outer membrane similar to that observed with TvTom40-2 (Fig 5B). A Pearson correlation coefficient displayed the highest degrees of colocalisation with TvTom40-2 for Tom46 (77%) and Tom22-like protein (63%). Decreasing degrees of colocalisation with TvTom40-2 were observed for Tom36 (46%), Homp19 (26%), and Homp38 (17%). These experiments showed that all the selected TA proteins reside in the hydrogenosomal outer membrane.

### TA proteins and Sam50 associated with TvTom40-2 are present in high molecular weight complex

**Fig 6:**
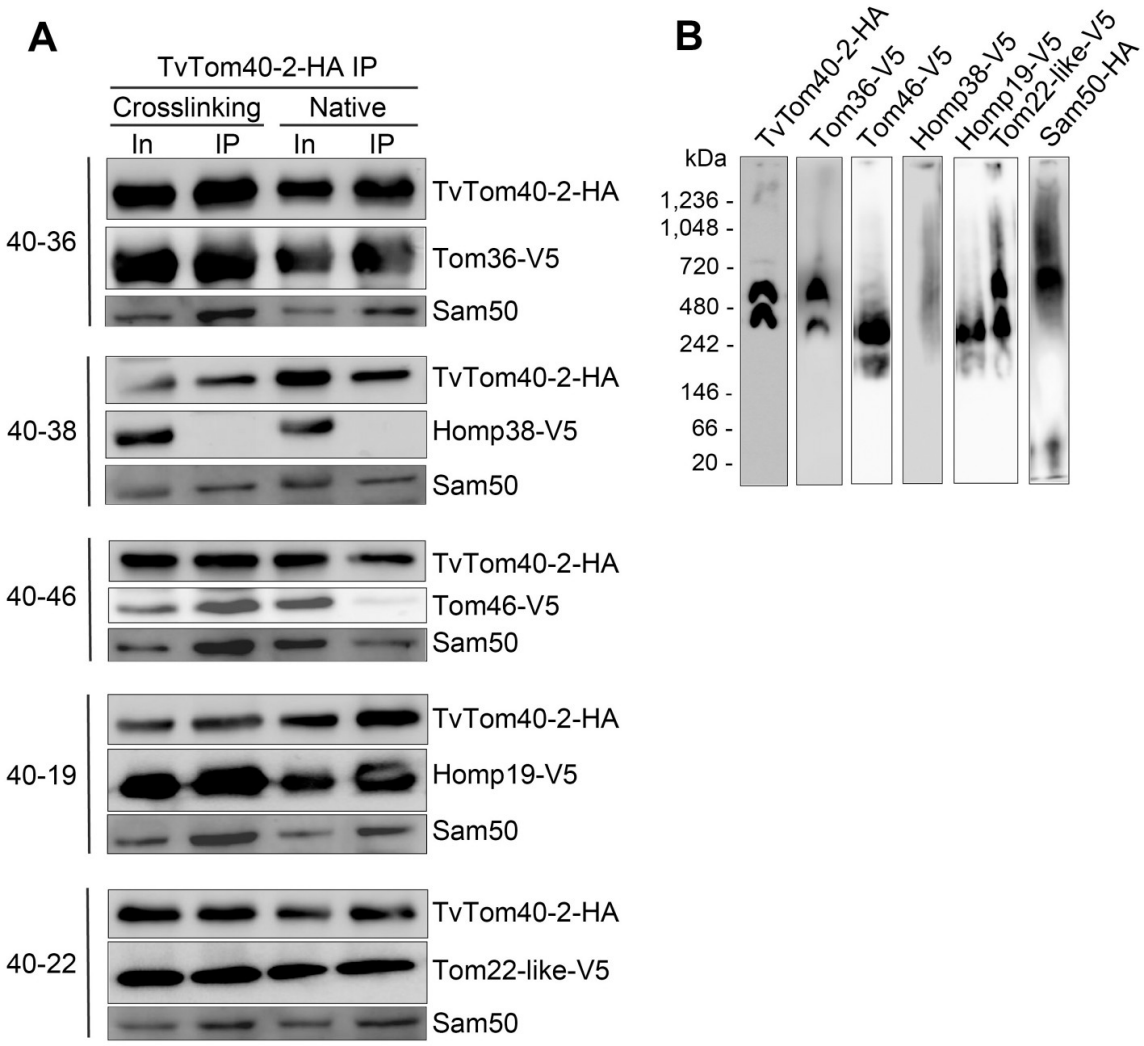
TA proteins and Sam50 associated with TvTom40-2 are present in a high molecular weight complex. (A) Digitonin-lysed extracts of hydrogenosomes isolated from the recombinant strains expressing both HA-tagged TvTom40-2 and one of the V5-tagged proteins, Tom36, Tom46, Homp38, Homp19, or Tom22-like protein were subjected to IP using α-HA antibody. Eluates from the IPs were probed for the presence of HA-tagged TvTom40-2, V5-tagged candidate proteins, and Sam50 under both crosslinking and native conditions using α-HA, α-V5, and polyclonal α-Sam50 antibodies, respectively. (B) BN-PAGE immunoblots of digitonin-lysed hydrogenosomal extracts from the strains expressing HA-tagged and V5-tagged proteins as indicated. BN-PAGE, blue native PAGE; HA, human influenza hemagglutinin; Homp, hydrogenosomal outer membrane protein; In, input; IP, immunoprecipitation; Sam, sorting and assembly machinery; TA, tail-anchored; TOM, translocase of the outer membrane; TvTom, *T*. *vaginalis* TOM.

To obtain further support for the association of identified TA proteins and Sam50 with the TvTOM complex, TvTom40-2-HA was pulled down from hydrogenosomes isolated from the double transfectants, and the samples were probed for V5-tagged proteins and Sam50 via immunoblotting using α-V5 and polyclonal α-Sam50 antibodies, respectively. Under crosslinking conditions, TvTom40-2 pulled down Tom36, Tom46, Homp19, and Tom22-like protein, while under native conditions, we observed a strong signal for Tom36, Homp19, and Tom22-like protein and a weaker signal for Tom46 (Fig 6A). Homp38 was not co-immunoprecipitated from the double transfectant under these conditions. On the other hand, Sam50 was detected in all samples analysed (Fig 6A). Furthermore, to validate whether the TvTom40-2-associated proteins are present in the high–molecular-weight complexes, hydrogenosomes isolated from the recombinant strains were subjected to BN-PAGE and immunoblotted with corresponding antibodies. Both Tom36 and Tom22-like protein migrated in 570 kDa and 330 kDa complexes. Tom46 and Homp19 migrated only in a 330 kDa complex, while Homp38 did not appear to be present in any high molecular weight complex. TvTom40-2, used as a reference, migrated at 570 kDa and 330 kDa under the same conditions when immunodecorated with α-HA antibody (Fig 6B). HA-tagged Sam50 migrated at 570 kDa and 55 kDa, which corresponded to the high molecular weight of TvTOM complex and to Sam50 monomer, respectively (Fig 6B). These results confirmed the association of Tom36, Tom46, Homp19, Tom22-like, and Sam50 with TvTom40-2, and their ability to incorporate into high molecular complexes.

### TvTom40-2 is involved in hydrogenosomal protein import

**Fig 7:**
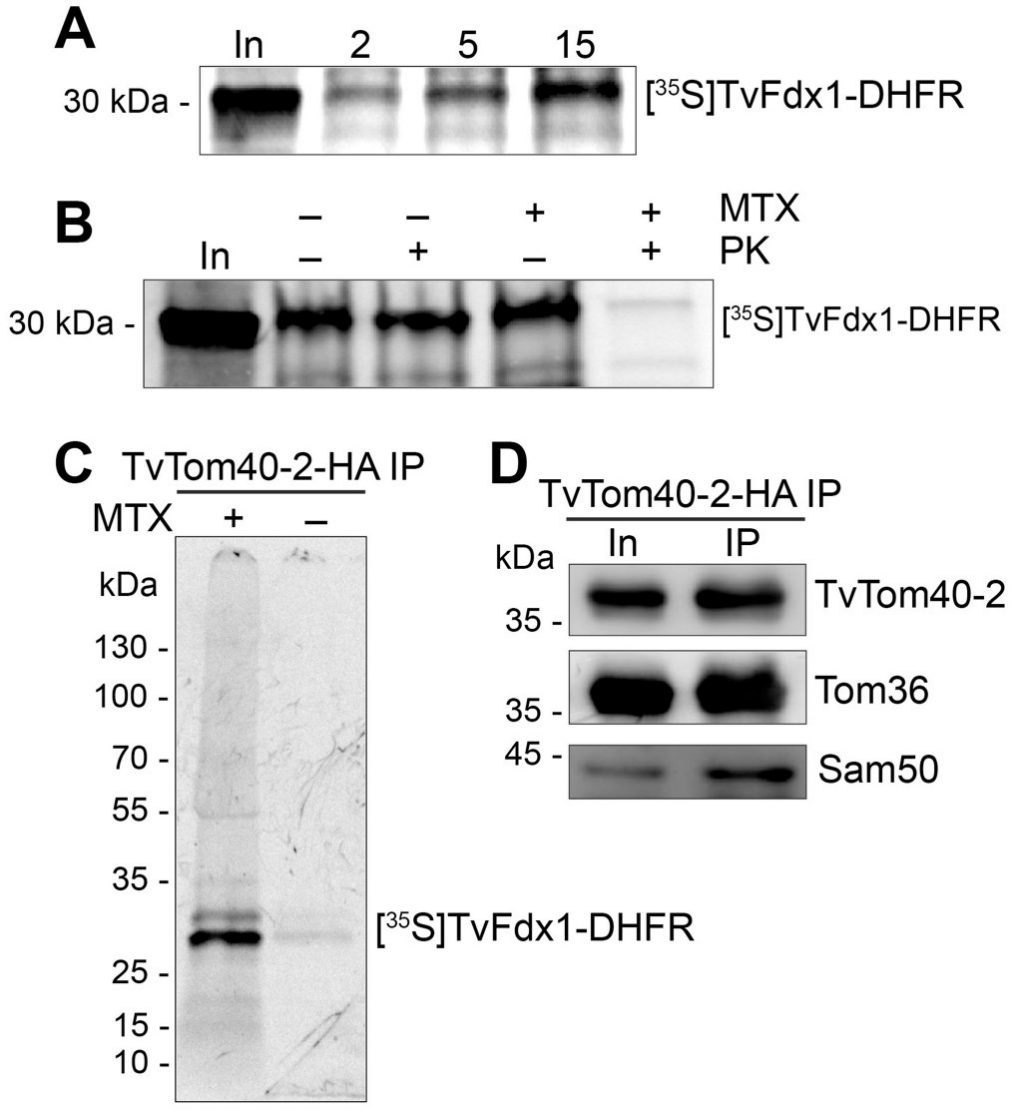
TvTom40-2 is involved in hydrogenosomal protein import. (A) Autoradiograph showing a time-dependent in vitro import of ^35^S-Met-labeled TvFdx1-DHFR into hydrogenosomes. (B) Autoradiograph showing the in vitro import of ^35^S-Met-labeled TvFdx1-DHFR into hydrogenosomes in either the absence (−) or the presence (+) of MTX, followed by proteinase K (+) treatment. (C) Autoradiograph showing the eluates for the TvTom40-2-HA coIP following the in vitro import of ^35^S-Met-labeled TvFdx1-DHFR into hydrogenosomes isolated from a strain expressing both TvTom40-2-HA and Tom36-V5 either in the presence (+) or the absence (−) of MTX. (D) Immunoblot of the same eluates as in panel C using α-HA, α-V5, and α-Sam50 antibodies. coIP, co-immunoprecipitation; DHFR, dihydrofolate reductase; Fdx, ferredoxin; HA, human influenza hemagglutinin; In, input; MTX, methotrexate; PK, proteinase K; Sam, sorting and assembly machinery; TOM, translocase of the outer membrane; TvTom, *T*. *vaginalis* TOM.

To demonstrate that the predicted TvTom40-2 participates in hydrogenosomal protein import, we performed an in vitro protein import and coIP assay. As an import substrate, we used the hydrogenosomal matrix protein ferredoxin (TvFdx1), which has an NTS fused to *Escherichia coli* dihydrofolate reductase (DHFR) at the C-terminus. TvFdx1-DHFR was synthesised in vitro in the presence of [^35^S]-methionine. Under standard in vitro import conditions, using hydrogenosomes isolated from the double-transfected TvTom40-2-HA/Tom36-V5 strain, TvFdx1-DHFR was imported into hydrogenosomes, which was confirmed by a protease protection assay. The autoradiograph showed a time-dependent import of TvFdx1-DHFR (Fig 7A). Next, in vitro import assay was performed in the presence of methotrexate, which is known to cause the folding of DHFR and therefore arrests the translocating protein at the mitochondrial protein import site [33]. As expected, TvFdx1-DHFR was arrested at the hydrogenosomal outer membrane, and the exposed region was degraded when the hydrogenosomes were treated with proteinase K (Fig 7B). Finally, to prove that TvTom40-2, Tom36, and the substrate are present in the same complex, we performed in vitro import assay for TvFdx1-DHFR either in the presence or absence of methotrexate, crosslinked the interacting proteins, and immunoprecipitated the complex via TvTom40-2-HA. Autoradiography of the eluted sample revealed the presence of arrested TvFdx1-DHFR associated with the complex when methotrexate was added (Fig 7C). The two bands present on the autoradiograph (lane 1) correspond to TvFdx1-DHFR (30 kDa) and its proteolytically cleaved product (29 kDa) most likely. Immunoblot analysis of the complex confirmed the presence of TvTom40-2 and Tom36 in the same sample (Fig 7D). No substrate signal was observed when methotrexate was omitted from the reaction mixture (Fig 7C). These results demonstrate that TvFdx1-DHFR was imported into hydrogenosomes in an unfolded state and the arrested TvFdx1-DHFR was associated with TvTom40-2 and Tom36.

### Tom36 and Tom46 can bind to hydrogenosomal preproteins

**Fig 8:**
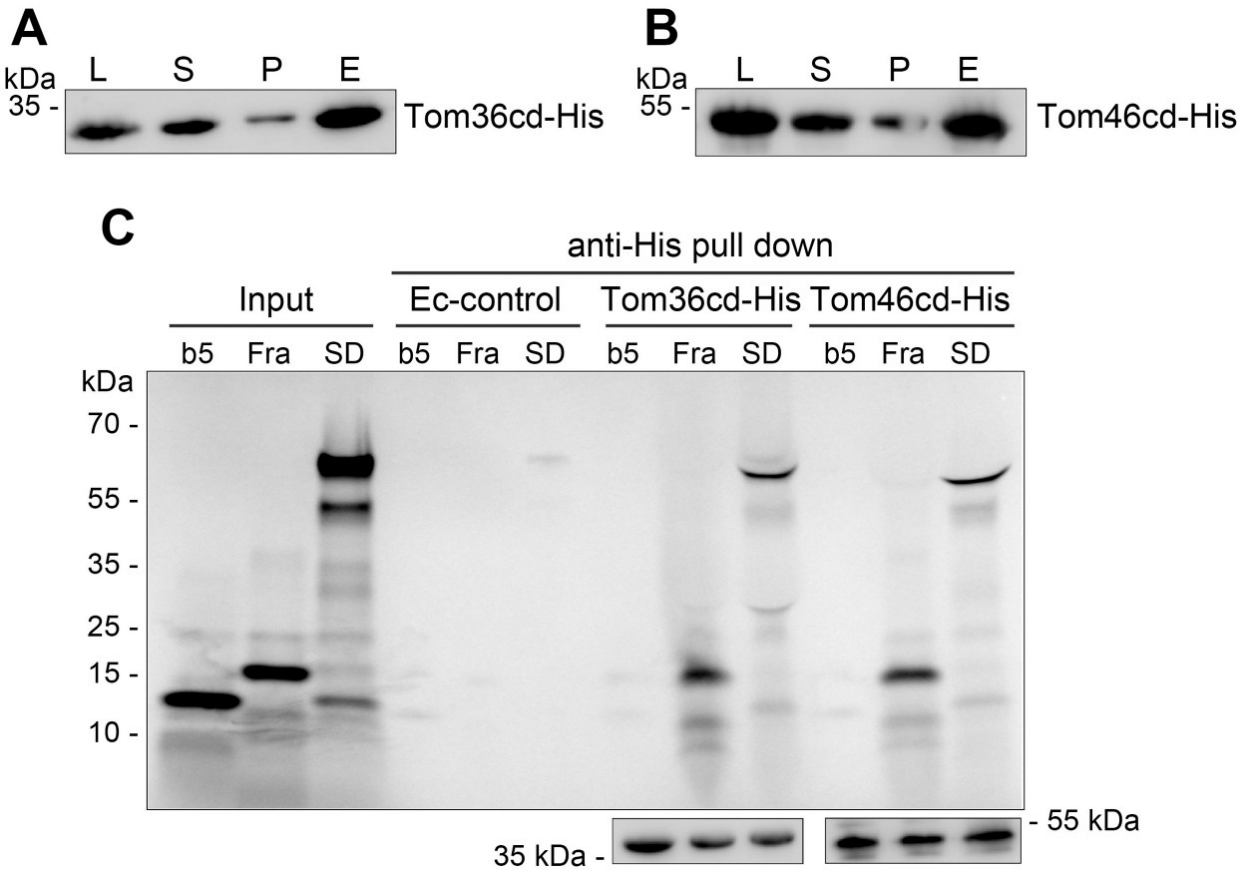
Tom36 and Tom46 can bind to hydrogenosomal preproteins. (A, B) Expression and coupling of His-tagged Tom36cd and Tom46cd to Ni-NTA agarose beads. *E*. *coli* cells expressing Tom36cd-His (panel A) or Tom46cd-His (panel B) were lysed (L; 2.5%), the lysate was centrifuged, and the supernatant with soluble proteins (S; 1%, input for the pull-down experiments) and pellet (P; 1%) fractions were obtained. The supernatant fraction was incubated with Ni-NTA agarose beads, and bound proteins were eluted (E; 5%) and probed on immunoblots using α-His antibody. (C) Binding assay. Proteins were pulled down from control *E*. *coli* or from cells expressing Tom36cd-His or Tom46cd-His using Ni-NTA agarose beads. The radiolabelled proteins cytochrome b5, frataxin, and αSCS-DHFR were incubated with various Ni-NTA agarose beads, and the His-tagged proteins were eluted using a buffer containing 500 mM imidazole. The samples were analysed by SDS-PAGE and autoradiography. The top panel shows an autoradiograph for the input radiolabelled proteins (Input; 10%) and the eluted fractions (20%). The bottom panel shows immunoblots using α-His antibody for Tom36cd-His and Tom46cd-His pull-down eluates (2.5%) from the binding assays. αSCS, α-subunit of succinyl CoA synthetase; b5, cytochrome b5; CoA, coenzyme A; DHFR, dihydrofolate reductase; Ec-control, control *E*. *coli*; Fra, frataxin; His, histidine; Ni-NTA, Ni-nitrilotriacetic acid; SD, αSCS-DHFR; SDS-PAGE, sodium dodecyl sulphate-PAGE; Tom, translocase of the outer membrane.

Because both Tom36 and Tom46 interact with TvTom40-2, are present in high–molecular-weight complexes, carry TPR-like domains and Hsp20-like chaperone domain that are involved in protein–protein interactions, and are paralogues, we selected these proteins as receptor candidates. To test whether they can bind to hydrogenosomal proteins, we performed in vitro binding assay. The cytosolic domain of Tom36 (Tom36cd, residues 1–308) and Tom46 (Tom46cd, residues 1–402) were expressed with a C-terminal polyhistidine (His) tag in *E*. *coli* BL21 (DE3) strain, respectively, and coupled with Ni-nitrilotriacetic acid (Ni-NTA) agarose beads (S4 Fig and Fig 8A and 8B). Beads preincubated with untransformed *E*. *coli* lysate were used as a negative control. A cytosolic protein cytochrome b5 was used as a negative control. Radiolabelled precursors of two hydrogenosomal matrix proteins, frataxin and the α-subunit of succinyl coenzyme A (CoA) synthetase (αSCS), with the latter fused to DHFR at the C-terminus (αSCS-DHFR), were incubated with Tom36cd-His or Tom46cd-His coupled with or mock-treated beads for 1 hour. Then, the His-tagged proteins with the bound substrates were eluted with imidazole. The eluate from the Tom36cd-His and Tom46cd-His binding assay showed the presence of two radiolabeled proteins, frataxin and αSCS-DHFR (Fig 8C, top panel). The cytosolic cytochrome b5 was not observed to be bound to either Tom36cd-His or Tom46cd-His (Fig 8C, top panel). Furthermore, the eluates were immunoblotted with anti-His antibody to verify the presence of His-tagged proteins (Fig 8C, bottom panel). These experiments indicate that the cytosolic domain of Tom36 and Tom46 can bind hydrogenosomal preprotein substrates.

### The TvTOM forms three protein translocation channels and has a unique skull-like structure

**Fig 9:**
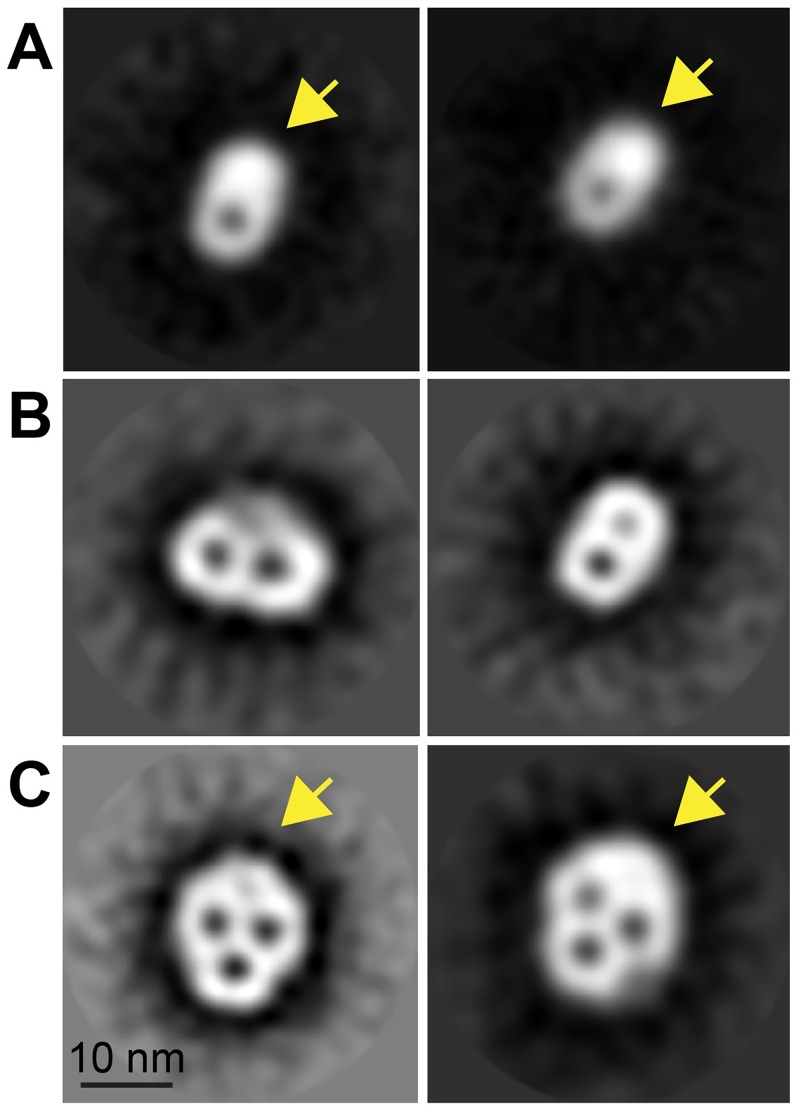
Electron microscopy analysis of the isolated TvTOM. Gallery of TvTOM class averages resulting from 2D classifications. Two class averages for TvTOM with (A) one pore at 24 Å resolution from 532 particles (left) and at 21 Å resolution from 517 particles (right); (B) two pores at 26 Å resolution from 348 particles (left) and at 31 Å resolution from 222 particles (right); and (C) three pores at 34 Å resolution from 298 particles (left) and at 21 Å resolution from 327 particles (right). Arrow indicates the additional mass. Scale bar, 10 nm. 2D, two-dimensional; TOM, translocase of the outer membrane; TvTom, *T*. *vaginalis* TOM.

The diversity of TvTom40 paralogues and the presence of unusual components in the TvTOM complex prompted us to investigate the structure of the TvTOM complex via electron microscopy analysis. The hydrogenosomal TOM complex was purified from *T*. *vaginalis* expressing TvTom40-2-HA under native conditions. The isolated hydrogenosomes were solubilised with digitonin to release the complex, and then the TvTOM complex was purified by IP using α-HA antibody coupled to Dynabeads and negatively stained for electron microscopy. The identity of the HA-tagged TvTom40-2 in the IP eluate was verified by immunoblotting and silver staining (S5A and S5B Fig). The unprocessed electron micrographs mainly showed particles composed of ring-shaped structures with one, two, or three centers of stain accumulation (representative micrograph in S5C Fig). These stain-filled openings are interpreted as pores, each of which represents one channel of the protein translocase. A total of 10,038 particles were selected from 650 micrographs for further processing. Two-dimensional (2D) classification with 3,412 particles (34% of 10,038 particles) resulted in class averages representing TvTOM with one, two, or three pores of resolution between 21 and 34 Å (Fig 9A–9C). TvTOM with one or two pores were the most prominent, accounting for 35% (*n* = 1,175) and 40% (*n* = 1,377), respectively, while TvTOM with three pores accounted for 25% (*n* = 860). The single-pore particles were oval, 70 × 125 Å in size with an eccentric pore placement. Two-pore particles were oval or triangular and 140 × 100 Å in size. The particles with three pores were skull-shaped and measured 150 × 175 Å in size, although a fourth spot of stain accumulation with a low contrast was observed in one of the class averages (Fig 9C). A single translocation channel measured 70 Å in diameter, and the inner pore size of the channel measured 25–30 Å. The distance between two pore centers measured 50–60 Å. The most striking difference from the yeast TOM is the presence of an extra density, measuring 50 Å in diameter observed in most classes of single-, double-, and triple-pore TvTOM particles, suggestive of a subunit(s) interacting with the peripheral part of the channel formed by TvTom40.

### Conserved core components and lineage-specific peripheral components of TOM complex in Excavata

**Fig 10:**
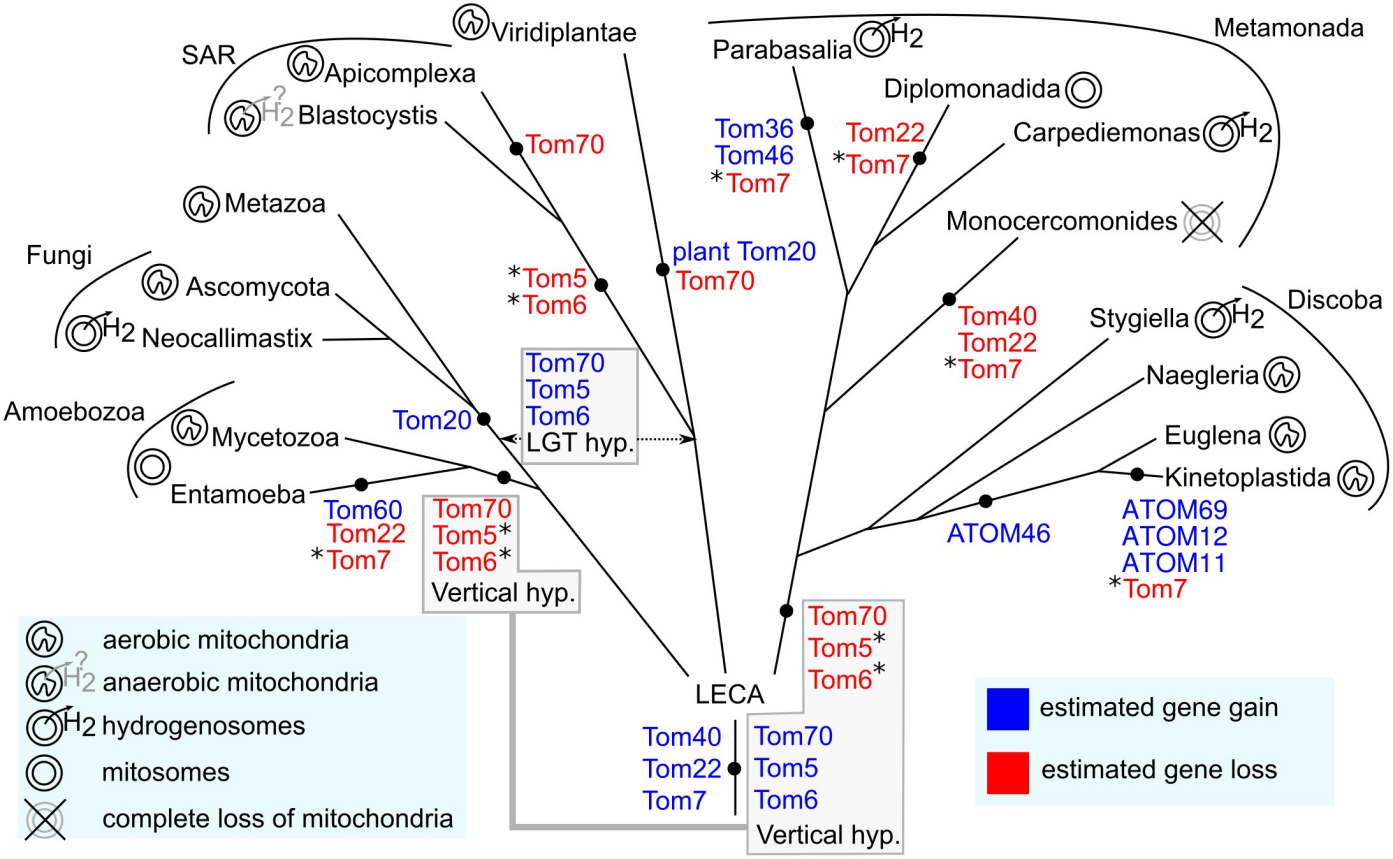
Phylogenetic scheme showing the gain and loss of TOM components across eukaryotic groups. Vertical gene transfer and LGT hypotheses for Tom5, Tom6, and Tom70 are in the boxes. Asterisk indicates small TOM subunits that were not identified; however, failure to identify them needs to be considered with caution. The relationships between the eukaryotic lineages are based on the recent phylogenetic results that employed concatenated gene data sets [42]. ATOM, archaic translocase of the outer membrane; LGT, lateral gene transfer; SAR, Stramenopiles, Alveolata and Rhizaria; TOM, translocase of the outer membrane.

Conservation of Tom40 and Tom22, and the identification of two novel peripheral components with Hsp20 and TPR domains (Tom36 and Tom46) suggest a peculiar evolutionary history for TvTOM complex. Therefore, we searched for orthologues of TOM components using a local HMM in selected genomes across different eukaryotic supergroups, with a focus on Excavata to estimate the conservation, gain, and loss of components (S3 Table and S3 Data). For our evolutionary scheme (Fig 10), we adapted a view that Excavata has two major sister groups: Metamonada, comprising anaerobic protists such as *T*. *vaginalis*, and Discoba, comprising *T*. *brucei* [34,35], although an alternative placement of Metamonada has been suggested [36]. Our phylogenomic profiling supported the current view that at least Tom40 and Tom22 are conserved in all eukaryotes and might have been present in the TOM complex of LECA (Fig 10). The only exception is *Monocercomonoides* sp., which has completely lost mitochondria including all genes coding for TOM and TIM components [37] (S3 Table and Fig 10). Support for Tom7 was less clear because neither *T*. *vaginalis* nor *T*. *brucei* seems to possess Tom7 (S3 Table and Fig 10). However, we took advantage of the available genome sequences of some free-living excavates [38–40] and identified putative Tom7 orthologues in *Carpediemonas membranifera* of Metamonada, and *Euglena gracilis* and *Stygiella incarcerata* of Discoba lineages (S3 Table and Fig 10). As expected, our searches showed that Tom20 and plant Tom20 were most likely gained independently in Opisthokonta and Viridiplantae, respectively, and their orthologues are not present in other lineages, including Excavata (S3 Table and Fig 10). The evolutionary history of Tom70, Tom5, and Tom6 is more complex. All three components have been found in opisthokonts, while only Tom5 and Tom6 are present in Viridiplantae. Conversely, in the supergroup Stramenopiles, Alveolata and Rhizaria (SAR), which is related to Viridiplantae [34], Tom5 and Tom6 are absent, whereas Tom70 was reported in *Blastocystis*, other SAR species, and the haptophyte *Emiliania huxleyi* [41] (S3 Table and Fig 10). In our searches, none of these three components have been identified in both Excavata and Amoebozoa (S3 Table and Fig 10). The most puzzling aspect is the appearance of unique peripheral TOM components in the Excavata group. The searches for proteins with the same domain structure as Tom36 (Hsp20-TPR-TMD) in the available genome of 11 excavates and in the genome of selected organisms from other eukaryotic supergroups revealed the presence of homologous proteins only in *Tritrichomonas foetus*, a close relative of *T*. *vaginalis* (Parabasalia lineage), in kinetoplastids, and interestingly, in a fungus *Neocallimastix californiae* (S3 Table and Fig 10).

**Fig 11:**
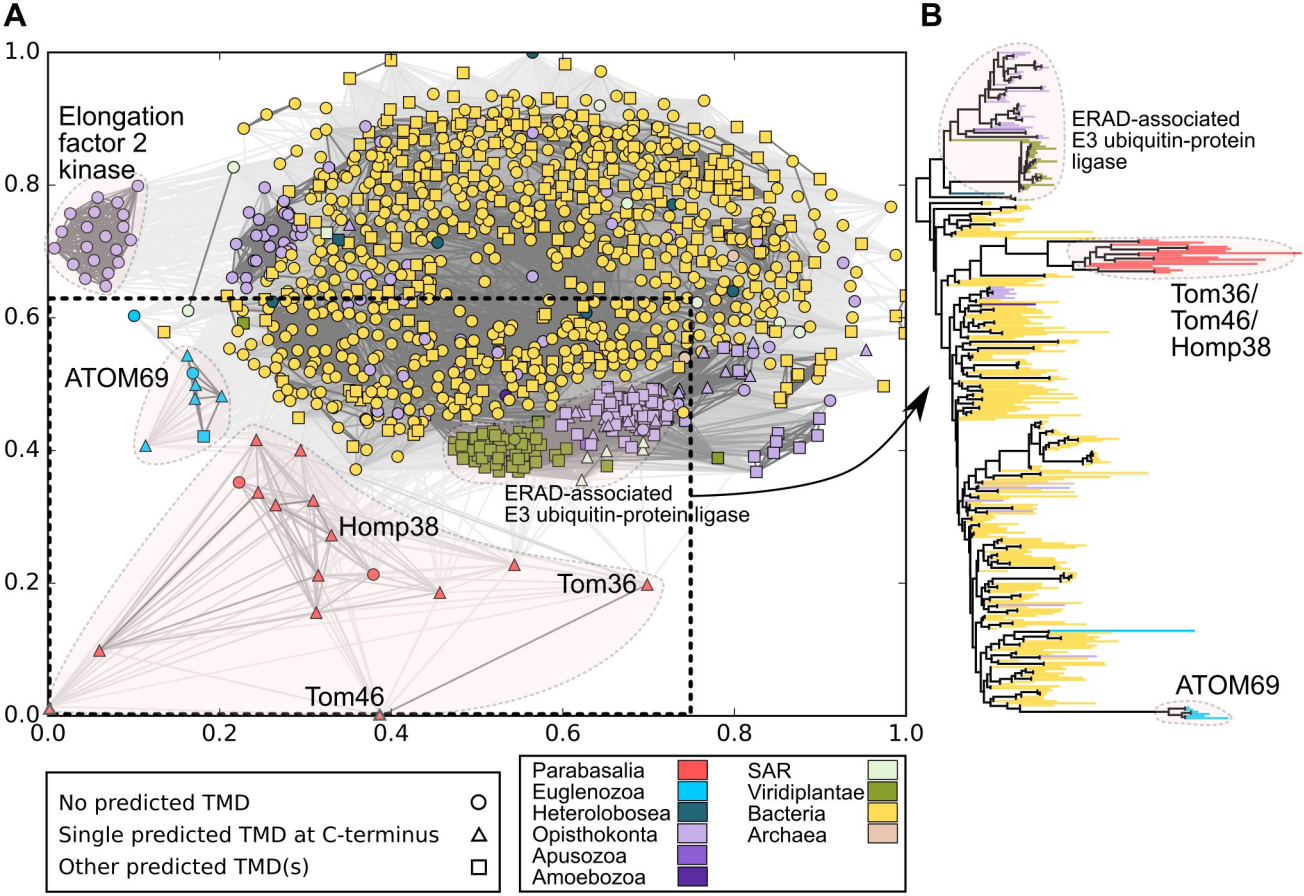
Relationship between Tom36/Tom46 and ATOM69. (A) CLANS similarity network for 1,114 homologues of Tom36 and ATOM69. The proteins from different eukaryotic and prokaryotic lineages are color coded. The prediction of the TMD using TMHMM is indicated by point shapes. For clarity, only 20% of the strongest connections between the proteins are shown in grey lines. The sequences and their coordinates for all the 1,114 proteins are given in S4 Data. Sequences within the marked rectangle were selected for the phylogeny. (B) Phylogeny of the TPR domains of Tom36, Tom46, ATOM69, and other related TPR proteins. The tree was constructed with IQ-TREE version 1.6.7 using the LG + I + G4 model and 10,000 ultra-fast bootstrap replicates. The sequences from different eukaryotic lineages and bacteria are color coded (418 taxa and 179 sites) (S5 Data). An enlarged version of the phylogenetic tree with accession numbers of taxa is shown in S6 Fig. ATOM, archai translocase of the outer membrane; CLANS, cluster analysis of sequences; ERAD, endoplasmic reticulum-associated protein degradation; Homp, hydrogenosomal outer membrane protein; SAR, Stramenopiles, Alveolata and Rhizaria; TMD, transmembrane domain; TMHMM, transmembrane helices hidden Markov model; Tom, translocase of the outer membrane; TPR, tetratricopeptide repeat.

Next, we performed homology searches using Tom36 or ATOM69 as queries against the National Center for Biotechnology Information (NCBI) nonredundant protein database regardless of the domain composition that resulted in a data set of 299 eukaryotic, 810 bacterial, and 5 archaeal sequences that were analysed using CLuster ANalysis of Sequences (CLANS) algorithm [43] (Fig 11A and S4 Data). Tom36 and Tom46 formed a cluster together with 10 other *T*. *vaginalis* and four *T*. *foetus* homologues (Fig 11A). All these homologues share Hsp20-TPR domains, two of them without any predicted TMD. A distinct cluster included seven ATOM69 homologues found in kinetoplastids that included dixenic, monoxenic, and free-living species (Fig 11A). The other clusters were formed by various TPR proteins, including elongation factor 2 kinase and endoplasmic reticulum-associated protein degradation (ERAD)-associated E3 ubiquitin-protein ligase (Fig 11A). The largest cluster predominantly contained bacterial proteins (Fig 11A). The formation of distinct clusters for Hsp20-TPR-TMD proteins of trichomonads and kinetoplastids suggests that Tom36/Tom46 and ATOM69 may have evolved independently in their respective lineages (Fig 11A). This view is supported by our phylogenetic analysis, in which Tom36/Tom46 and ATOM69 form two separate branches that are interleaved by a large bacterial group (Fig 11B).

## Discussion

In spite of the fundamental role of mitochondrial translocases for the function and evolution of the eukaryotic cell, our experimental knowledge of the TOM complex is limited to a few model organisms, and direct visualisation of the TOM complex has only been achieved in two fungi, *S*. *cerevisiae* and *N*. *crassa* [14,15]. To extend our knowledge on TOM diversity in eukaryotes, we isolated and characterised the TOM complex from hydrogenosomes, an anaerobic form of mitochondria in *T*. *vaginalis*. In the present study, we have demonstrated the function of a highly divergent pore-forming TvTom40-2 and identified a protein that has limited homology with Tom22. The other components of TvTOM include three TA proteins with no orthologues in the fungal TOM complex. Furthermore, TvTOM seems to be tightly associated with Sam50 for a more efficient β-barrel biogenesis.

Electron microscopic visualisation of the TvTOM complex revealed interesting similarities and differences when compared with the TOM complex in fungi. Most observed TvTOM particles displayed two pores, which in fungi represent the TOM core complex, or particles with three pores, corresponding to the holo complex. The distance between two pore centers, the inner pore diameter, the single translocation channel diameter, and the size of the particles with two pores are similar to those determined for the TOM complex in fungi [15,20]. The appearance of single-pore particles could more likely be either a result of the dissociation of holo complexes during experimental procedures [20,44] or stable assembly intermediates. A striking deviation from known TOM models is the presence of an extra density in the single-, double-, and triple-pore particles, providing a skull-like shape to the TvTOM holo complex. Based on coIP-MS analysis, it can be speculated that the extra mass may contain the identified β-barrel proteins Sam50 or Hmp35. In yeast, the TOM and SAM complexes form a labile supercomplex that allows coupling of the translocation of the Tom40 precursor through TOM and its insertion into the OMM via SAM [17]. It has been suggested that Sam50 may account for the third pore in the yeast triplet-pore complex [15]. Cryo electron microscopy (Cryo-EM) has shown that the Sam50 monomer measures 50 Å [45], which is consistent with the size of the additional mass observed in TvTOM. BN-PAGE analysis showed that HA-tagged Sam50 migrated with the high–molecular-weight complex of TvTOM or as a monomer. The enrichment of TOM subunits, as well as Sam50 in the reciprocal coIPs, supports a tight TOM-Sam50 association in hydrogenosomes. Formation of the supercomplex in yeast is mediated by the N-terminal cytosolic domain of Tom22 and Sam37 [17,18]. In trichomonads, Sam37 has not been identified [28], and Tom22 has a short cytosolic domain. Therefore, if the observed association of TvTOM and Sam50 represents a functional complex, different protein–protein interactions are to be expected. Hmp35 is a β-barrel protein in the hydrogenosomal membrane with an unknown function that exists in a stable 300 kDa complex of Hmp35 oligomers [27]. This complex is too large to imply the formation of a complex with TvTOM.

The presence of a TOM complex with three pores observed in *T*. *vaginalis* strongly indicates that triplet-pore complex is the generic form of TOM in eukaryotes that was inherited from LECA. It has been proposed that the ancient TOM complex contained—in addition to Tom40—Tom22, which tethers Tom40s using its TMD, and a regulatory subunit Tom7 [4,12,16,46]. The Excavata group includes two major lineages, Metamonada and Discoba, represented by *T*. *vaginalis* and *T*. *brucei*, respectively. Investigations of *T*. *brucei* TOM complex initially suggested that Tom40 in kinetoplastids (ATOM40) might be a homologue of the bacterial Omp85-like protein [13]. However, profile-sequence searches found that ATOM40 belongs to the eukaryotic porin family [12,47]. Our analysis, with an extended sampling of Excavata—which included a Tom40 orthologue in *E*. *gracilis*, which shares a common ancestry with kinetoplastids—confirmed this view.

Previous sequence searches implied the absence of Tom22 in some excavates with reduced forms of mitochondria, including the hydrogenosomes of *T*. *vaginalis* [12]. However, due to its short sequence and low conservation [4,12,32], the identification of Tom22 might have been beyond the sensitivity of most search tools. Our sensitive, structure-based HMM search identified a short 6 kDa Tom22-like protein as a potential candidate. This protein is tightly associated with TvTom40-2 in the hydrogenosomal outer membrane and is present in both high molecular weight complexes (570 and 330 kDa). Tom22-like protein contains a conserved TMD motif, including invariable tryptophan and proline residues, and a short cytosolic N-terminal (*cis*) domain similar to the 9 kDa Tom22 orthologue, Tom9 in higher plants, the 8 kDa apicomplexan Tom22, and the kinetoplastid Tom22 orthologue, ATOM14 [4,31,32]. The long acidic extension of the *cis* domain evolved only in opisthokonts that interacts with lineage-specific Tom20 and Tom70 [4], and therfore its absence in Tom22-like protein is not surprising. Most Tom22s contain an IMS-localised acidic (*trans*) domain that interacts with the substrate and enhances its transfer to Tim50 in the TIM23 complex [19]. Tom22-like protein identified here lacks the *trans* domain, which may reflect the absence of Tim50 in *T*. *vaginalis* [28]. In addition to *T*. *vaginalis* and *T*. *brucei*, we retrieved Tom22 orthologues from members of both Metamonada and Discoba in support of its presence in Excavata common ancestor.

Tom7 has not been identified in parabasalids, diplomonads, and in kinetoplastids. A fusion protein with limited sequence similarity to Tom7 and Tom22 has been reported in *Naegleria* species [12]. Importantly, Tom7 orthologues appears to be present in free-living members of both Excavata lineages, in *C*. *membranifera* (Metamonada), and *E*. *gracilis* and *S*. *incarcerata* (Discoba). This suggests that the absence of Tom7 might be a result of a secondary loss, and if so, it happened independently in certain lineages of both Metamonada and Discoba. However, failure to identify small Toms—Tom7 as well as Tom5 and Tom6—needs to be tread with caution. Their sequences are very short and might be highly divergent, particularly in parasitic lineages and those with reduced forms of mitochondria, which can hamper their identification. Collectively, our results suggest that the triplet-pore form of the TOM complex constituted the ancestral form of TOM in LECA.

Functional studies of TvTom40-2 using a DHFR-methotrexate system demonstrated that hydrogenosomal preprotein binds to TvTom40-2 and subsequently is imported into the hydrogenosomal matrix in an unfolded or loosely folded state, a feature that is conserved in mitochondria [33]. Of note, *T*. *vaginalis* has at least seven TvTom40 paralogues that are all expressed [10]. CoIP-MS analysis revealed that TvTom40-2 is associated with five other paralogues, and therefore various combinations of TvTom40 paralogues appear to be present in a single TOM complex, as observed in the rat TOM complex, in which two Tom40 isoforms interact with each other [48]. Further, we asked whether the hydrogenosomal TvTom40-2 could be integrated and can function in the yeast OMM. Despite low amino acid sequence conservation between TvTom40-2 and yeast orthologue, heterologous expression of TvTom40-2 in yeast resulted in its localisation in the OMM and the formation of a 230 kDa complex. This finding is consistent with the recent investigation of the targeting signal in β-barrel proteins, wherein the signal appears not to be encoded in a conserved linear amino acid sequence but is embedded in the structure of a β-hairpin motif [49]. Such a targeting signal was likely inherited from bacterial β-barrel proteins and remains conserved across all eukaryotic lineages, as supported by our experiment. As observed via protease protection assay, the topology of TvTom40-2 both in hydrogenosomes and mitochondria was similar. Interestingly, TvTom40-2 was able to very partially substitute yeast Tom40, indicating that at least some proteins were imported into yeast mitochondria through TvTom40-2. It is of note that some yeast mitochondrial proteins were imported into hydrogenosomes of *T*. *vaginalis* regardless of the presence or absence of NTS [26]. Based on this, it was proposed that the hydrogenosomal Tom40 is able to recognise unspecified ITSs conserved in the proteins of mitochondrial ancestry [26].

The key question is whether the TvTOM complex in hydrogenosomes consists of only core subunits or whether there any peripheral TOM subunit(s) that contribute to the import of proteins. This is expected because both NTS- and ITS-dependent protein targeting to hydrogenosomes have been demonstrated [24–26]. However, our HMM searches confirmed the absence of known TOM receptors Tom20 and Tom70 in excavates. These receptors either evolved only in certain eukaryotic lineages (Tom20) or were present in LECA (Tom70) as hypothesised here and by others [41]. To identify yet unknown peripheral TvTOM subunits, we performed proteomic analyses of the isolated TvTOM complex that indicated the presence of three TA proteins, in addition to Tom22-like protein. Two of them, Tom36 and Tom46, possess Hsp20-TPR-TMD architecture, which is similar to *T*. *brucei* receptor ATOM69. Indeed, we observed that Tom36 and Tom46 could bind to two hydrogenosomal preproteins, frataxin and αSCS, through binding assay. Tom36, Tom46, and ATOM69 are similar to yeast Tom70 with respect to the presence of TPR domains. The proximal TPR set of Tom70 interacts with Hsp90 [50] and may have an analogous function with the Hsp20 domain in Tom36, Tom46, and ATOM69 [11]. Of note, only Tom36 was tightly associated with TvTom40-2 and was detected in both high–molecular-weight complexes, whereas Tom46 appears to be loosely associated because it appeared only in the 330 kDa complex. This is similar to the loose association of Tom70 with the TOM complex that was reported in *N*. *crassa* [20] and the absence of Tom70 in the 550 kDa TOM complex in *S*. *cerevisiae* [14]. The third protein, Homp19, is unique to *T*. *vaginalis*, and neither HHpred nor PfamA searches identified any known functional domains.

It is tempting to speculate that the subunits with similar Hsp20-TPR-TMD architecture in both *T*. *vaginalis* and *T*. *brucei* evolved from a common excavate ancestor. However, our phylogenetic profiling of Hsp20-TPR-TMD proteins revealed that they were present exclusively in parabasalids and kinetoplastids but absent in the basal lineages, *S*. *incarcerata* (Discoba), *Naegleria gruberi* (Discoba), and *C*. *membranifera* (Metamonada). Therefore, such a distribution is more consistent with independent gains in parabasalid and kinetoplastid lineages. This is also supported by our cluster analysis and phylogeny of TPR domains, in which Tom36/Tom46 and ATOM69 displayed a polyphyletic origin. This finding is interesting considering the recent phylogenetic studies that challenged the monophyletic origin of Excavata [35,36]. Although the phylogenetic analysis of Excavata—including long-branch members such as trichomonads—placed Metamonada as a sister group of Discoba, when long-branch representatives were excluded, these two groups separated [35]. Regardless of whether the origin of Excavata is monophyletic or polyphyletic, Tom36/Tom46 and ATOM69 most likely represent an example of convergent evolution rather than a diversification of a common ancestor.

In spite of the presence of Tom40 and Tom22 homologues, the hydrogenosomal TvTOM complex revealed considerable differences compared with the mitochondrial TOM complex. There are several constraints to be considered for the specific shaping of TvTOM. Hydrogenosomes are adapted to operate under anaerobic conditions, which resulted in a vast reduction of mitochondrial functions and, consequently, a reduction in the proteome from 1,000–1,500 proteins in mitochondria [51–53] to approximately 600 proteins in *T*. *vaginalis* hydrogenosomes [10,54]. In yeast, the positively charged NTS, forming an amphipathic α-helix, interacts with Tom20, the *cis* and *trans* domains of Tom22, and the presequence-binding groove of the Tim50 receptor during translocation across the OMM [55]. The positive charge of the NTS contributes to the membrane potential (Δψ)-driven import step through TIM23 [56]. However, hydrogenosomes have lost the inner-membrane–associated respiratory chain that generates Δψ, and this loss has possibly triggered the positive net charge of NTS to become dispensable. Indeed, most hydrogenosomal NTSs possess only a single positively charged residue [57], are considerably shorter, are not essential for preprotein import, and—in a number of matrix proteins—are not present. Thus, the import of these proteins is based on recognition of poorly understood ITSs [25,26,57]. These changes in the targeting signals are likely reflected by the modifications in TOM receptors, the loss of both Tom22 *trans* domain and Tim50, and the divergence of downstream import machinery [10]. Collectively, the adaptation to anaerobiosis and the loss of Δψ were critical constraints that may have allowed mutation, leading to the divergence of the TvTOM complex. Another reason for the divergence of TvTOM could be different evolutionary history of the lineage. Our finding of trichomonad Tom36 and Tom46 in Parabasalia and the phylogenomic profiling of TOM components supports the notion that the peripheral TOM subunits were added to the core components after the separation of the main eukaryotic lineages.

## Materials and methods

### Cell cultivation

*T*. *vaginalis* strain T1 (J. H. Tai, Institute of Biomedical Sciences, Taipei, Taiwan) and the recombinant strains were grown in Tryptone-Yeast extract-Maltose medium (TYM; pH 6.2) with 10% (v/v) heat-inactivated horse serum, without or with 200 μg/mL Geneticin 418 (Single transfectant), or with both 200 μg/mL Geneticin 418 and 40 μg/mL Puromycin (Double transfectant) at 37 °C. Recombinant *E*. *coli* strains were grown on Luria-Bertani medium with 100 μg/mL of Ampicillin at 37 °C. The yeast strains were grown either in liquid medium (SD-Leucine or SLac-Leucine) or on solid medium (SD-Leucine or YPG) at 30 °C. For drop dilution assays, cells were cultured to an OD_600_ of 1.0 and diluted 5-fold, followed by spotting 5 μL of each dilution on SD-Leu, SD-Leu supplemented with 2 μg/mL Dox, YPG, or YPG supplemented with 2 μg/mL Dox.

### Preparation of recombinant strains

The genes encoding TvTom40-2 (TVAG_332970) and Sam50 (TVAG_178100) were cloned into a pTagVag2 vector fused to a 2×HA tag at the C-terminus [58]. The genes encoding Tom36 (TVAG_277930), Tom46 (TVAG_137270), Homp38 (TVAG_190830), Homp19 (TVAG_283120), and Tom22-like protein (TVAG_076160) were cloned into a pTagVagV5 vector fused to a 2×V5 tag at the C-terminus [59]. The plasmids were transfected by electroporation [58] into either the WT strain or the strain expressing HA-tagged TvTom40-2. For studies in yeast, TvTom40-2 was cloned into a pYX142 vector (Novagen) fused to an HA tag at the C-terminus. The plasmid with no insert or plasmid encoding either HA-tagged TvTom40-2 or ScTom40 was transformed into yeast cells (WT strain W303α, tet-*TOM40*, *tom40*-25, and *tom40*-34) by lithium acetate method. The tet-*TOM40* yeast strain was constructed by inserting the tetracycline operator into the genome of WT strain, YMK120, upstream of *TOM40* ORF by homologous recombination, using an insertion cassette amplified from the plasmid pMK632 as described previously [60]. Yeast strains carrying temperature-sensitive alleles of *TOM40*, *tom40*-25, and *tom40*-34 were obtained from elsewhere [61]. The oligonucleotides used are listed in S4 Table.

### Bioinformatics

Tom40-like protein sequences from *T*. *vaginalis* were searched against the NCBI Conserved Domains database and the *S*. *cerevisiae* proteome or against Protein Data Bank (PDB) using the HHpred tool [62]. A Tom40-specific HMM was built using the HMMER3 hmmbuild module [63], with a set of 24 well-annotated Tom40 sequences (S1 Data) and was scanned against the *T*. *vaginalis* protein database on the HMMER3 jackhmmer tool with the default settings [64]. Human Tom22 and Tom7 sequences were searched against the NCBI nonredundant protein database using three PSI–Basic Local Alignment Search Tool (BLAST) iterations from different eukaryotic organisms. The alignments for Tom22 and Tom7 were constructed using MAFFT [65] with 447 (S6 Data) and 349 (S7 Data) sequences, which were used to build Tom22-specific and Tom7-specific HMMs, respectively, and were searched against the Trichomonas proteome database (www.trichdb.org) using HMMER3 [64].

The homologues of 14 TOM subunits were searched against the predicted proteomes of selected eukaryotes using HHsearch. The query alignments and their sources are given in S8 Data. The best hits were then checked for conserved domains using HHpred (https://toolkit.tuebingen.mpg.de/#/tools/hhpred) and were searched against the NCBI nonredundant protein database using BLAST. The transmembrane helices were predicted using TMHMM server version 2.0 (http://www.cbs.dtu.dk/services/TMHMM/) with a relaxed cutoff of 0.3. For CLANS [43], an extensive data set of Tom36 and ATOM69 homologues was prepared. Tom36 and ATOM69 protein sequences were used as queries to search against the NCBI nonredundant protein database using PSI–BLAST with two iterations, and the sequences with an e-value less than 0.1 were selected. Altogether, 1,114 sequences were used for CLANS, which was run with 10,000 iterations. The obtained 2D clustering data were processed to color-code taxonomies. The TMD was predicted using TMHMM with a relaxed cutoff of 0.3. A subset of 418 sequences from the data set was selected for the phylogenetic analysis of their TPR domains. The TPR domains were detected using HHsearch with TPR domains from the COG database (COG0790) as a query. Multiple sequence alignment was created with MAFFT [65], and the alignment was trimmed with BMGE [66], which resulted in 179 sites. The phylogenetic tree was constructed with IQ-TREE [67] using the LG + I + G4 model and 10,000 ultra-fast bootstrap replicates.

### Structural modeling

The model of TvTom40-2 was built using the *N*. *crassa* Tom40 structure (PDB ID 5o8o) as a template. The alignment was based on 140 Tom40 and VDAC sequences from a wide spectrum of eukaryotic organisms (S9 Data). The alignment was constructed by MAFFT, using the local pair alignment settings and 100 iterations [65] and later manually edited to reflect the secondary structure prediction of TvTom40-2 made by PSIPRED [68]. The three-dimensional (3D) structure model of TvTom40-2 was built using MODELLER 9v17 [69]. The quality of the final model was verified using ModFOLD 6 [70,71]. The electrostatic potential on the solvent-accessible surface of TvTom40-2 was calculated using APBS tool2 [72].

### Subcellular fractionation, protease protection assay, alkaline carbonate extraction, and immunoblotting

*Trichomonas* cells from a 1 liter culture were harvested and homogenised by sonication, and the subcellular fractions were isolated by differential centrifugation, as described previously [10]. Isolated hydrogenosomes (protein concentration 1 mg/mL) carrying either HA-tagged or V5-tagged proteins were washed to remove protease inhibitors and incubated for 30 minutes at 37 °C in isolation buffer (225 mM sucrose, 10 mM KH_2_PO_4_, 20 mM HEPES, 0.5 mM KCl, 5 mM MgCl_2_, and 1 mM EDTA [pH 7.2]) supplemented with either 100 μg/mL proteinase K enzyme (Roche Holding AG, Basel, Switzerland) or proteinase K with 0.5% Triton X-100. The incubation was terminated using 1 mM of phenylmethylsulfonyl fluoride (PMSF, Sigma Aldrich). Then, samples were analysed by immunoblotting using α-HA, α-V5, α-Fdx1, α-cytosolic malic enzyme, or α-αSCS antibody, followed by either α-mouse or α-rabbit antibody conjugated to peroxidase. The blot was developed using Amersham imager 600. Subcellular fractionation for yeast strains, and alkaline carbonate extraction and protease protection assay with isolated mitochondria were performed as described previously [73]. Proteins were separated by SDS-PAGE; immunoblotted with α-HA, α-HK, α-Fis1, or α-Aco antibody; and developed using an ECL system.

### Immunofluorescence and STED microscopy

The cells for immunofluorescence microscopy were processed as previously described [74]. Recombinant proteins were visualised using mouse α-HA and rabbit α-V5 antibodies, and Alexa Fluor 488 donkey α-mouse and Alexa Fluor 594 donkey α-rabbit antibodies (Thermo Fisher Scientific). The hydrogenosomal marker malic enzyme was detected by rabbit polyclonal antibody. The slides were mounted using Vectashield containing DAPI (4',6-diamidino-2-phenylindole) (Vector laboratories). The cells were examined with an Olympus Cell-R IX-81 microscope, and the images were processed using ImageJ. For STED, Abberior STAR 580 α-mouse and Abberior STAR 635p α-rabbit antibodies, along with Abberior TDE mounting medium, were used. STED images were acquired on a commercial Abberior STED 775 QUAD Scanning microscope (Abberior Instruments) equipped with a Nikon CFI Plan Apo Lambda objective (60× Oil, NA 1.40). Abberior STAR580- and STAR 635P-labeled proteins were illuminated by pulsed 561 nm and 640 nm lasers and depleted by a pulsed 775 nm STED depletion laser of the 2D donut. Fluorescence signal was filtered (Emission bandpasses: 605–625 nm and 650–720 nm; pinhole 40 μm) and detected on single photon counting modules, with time gates set to 0.8–8.8 ns. Images were scanned with a pixel size of 20 nm × 20 nm, with a 10 μs dwell time and in-line interleaved acquisition mode using the Imspector software. All images were deconvolved with Huygens Professional version software 17.04 using the Classic Maximum Likelihood Estimation algorithm.

### BN-PAGE

Isolated hydrogenosomes from the recombinant strains expressing tagged proteins were lysed with the native sample buffer (Life Technologies) containing either varying concentrations (1%–3%) of digitonin or 1% digitonin. The clarified extracts were electrophoresed on 3%–12% or 4%–16% NativePAGE bis-tris gel (Thermo Fisher Scientific), immunoblotted with either α-HA or α-V5 antibody, and developed by chemiluminescence. For BN-PAGE with yeast cells, isolated mitochondria from the strain with empty plasmid, or from strain expressing HA-tagged TvTom40-2, were lysed with lysis buffer containing 1% digitonin, and the clarified samples were electrophoresed on a 6%–13% native gel, immunoblotted with either α-HA or α-ScTom40 antibody, and developed using an ECL system.

### Crosslinking and native coIP

CoIPs were performed for the HA-tagged TvTom40-2 either with or without crosslinker using isolated hydrogenosomes from both WT and recombinant strains. For crosslinking, interacting proteins in hydrogenosomes (protein concentration 1 mg/mL) were crosslinked with 1 mM DSP (dithiobis(succinimidyl propionate); Thermo Scientific) for 30 minutes at 25 °C, excess DSP was quenched with 50 mM Tris (pH 7.5), and the hydrogenosomes were washed twice with isolation buffer. For coIP, the hydrogenosomes (protein concentration 1 mg/mL) were solubilised in MKG buffer (10 mM MOPS [3-(N-morpholino)propanesulfonic acid; pH 7], 50 mM potassium acetate, 10% glycerol, and EDTA-free cOmplete protease inhibitor cocktail [Roche]) containing 1% digitonin (Merck Millipore), and the clarified extract was incubated with Dynabeads (Thermo Fisher Scientific) coupled with α-HA antibody for 90 minutes on an overhead rotator at room temperature. The beads were washed thrice before elution with either SDS-PAGE buffer for crosslinking coIPs or elution buffer (MKG buffer with 0.25% digitonin and 1 mg/mL HA peptide, Thermo Fisher Scientific) for native coIPs. The coupling of α-HA antibody to the Dynabeads was performed according to the manufacturer’s instructions.

### LFQ-MS analysis

LFQ-MS was performed according to standard procedures as described previously [59]. To remove SDS from the crosslinking coIP eluates and to remove HA peptides from the native coIP eluates, samples were resuspended in 8 M urea and processed using a Filter Aided Sample Preparation (FASP) protocol, according to Wisniewski et al. [75]. The samples were digested with trypsin and the peptides obtained were subjected to liquid chromatography-MS. The MS/MS spectra obtained were searched against the *T*. *vaginalis* database (downloaded from Trichomonas Genome Resource [TrichDB; www.trichdb.org] containing 59,862 entries), the quantifications were performed with the label-free algorithms, and the data analysis was performed using Perseus 1.5.2.4 software. The MS data have been deposited to the ProteomeXchange consortium via the PRIDE [76] partner repository. The MS data were obtained from four independent coIP experiments for each immunoprecipitated protein.

### Isolation of the TvTOM complex, transmission electron microscopy, and data analysis

The TvTOM complex was purified under native conditions from hydrogenosomes isolated from the recombinant strain expressing C-terminal HA-tagged TvTom40-2 as described earlier. Five microliters of purified TvTOM complexes in solution was applied to copper electron microscopy grids (EMS200-Cu) covered with a 20 nm carbon film, which were glow discharged for 40 seconds with a 5 mA current prior to specimen application. Excess sample was removed after 1 minute by blotting (Whatman no. 1 filter paper) for 1 to 2 seconds, and the grid was immediately stained with 5 μL of 2% phosphotungstic acid for 1 minute 40 seconds and blotted to remove excess stain. A large data set of optimised, negatively stained specimen grids was acquired with a Tecnai F20 microscope (Thermo Fisher Scientific) operating at an accelerating voltage of 200 kV, with a FEI Eagle 4K CCD camera, at a magnification of 78,000× and a pixel size of 1.79 Å. Altogether, 1,000 images were acquired with defocus ranging from 2 to 5 μm. After quality inspection and determination of Contrast Transfer Function (CTF) parameters with the GCTF program [77], 650 micrographs were subjected to particle picking. Approximately 6,000 particles were manually picked from the first 200 micrographs with the e2boxer.py routine of the EMAN2 program [78] and subjected to three rounds of class averaging in Relion 1.4 [79], with 200, 150, and 100 classes, respectively. The box size was set to 192 pixels to accommodate higher-order multimers. This analysis resulted in a set of three representative class averages, which were low-pass filtered to 30 Å and used as templates for automated particle selection of the preselected set of 650 micrographs with the Gautomatch program. Altogether, 71,834 identified particles were subjected to five rounds of 2D classification in Relion with 200 classes, which reduced the data set to 10,038 particles. All 2D classifications comprised 40 iterations. The presented resolution of the class averages corresponds to the lowest SSNR value ≥1 indicated in the *model.star file resulting from the last iteration of the final 2D classification. The number of particles contributing to the class averages was also found in the *model.star files.

### In vitro protein import assay

The gene encoding Ferredoxin1 (TVAG_003900) was cloned into NEB PURExpress control vector fused to the DHFR gene (*E*. *coli*) at the C-terminus. Radiolabeled TvFdx1-DHFR was synthesised in vitro in the presence of L-[^35^S] methionine (MGP spol sro) according to the manufacturer’s instructions (NEB PURExpress in vitro protein synthesis kit). Cytoplasmic extract was prepared from the *T*. *vaginalis* strain T1 as described elsewhere [24]. For the time course experiment, the import assay was conducted in a 500 μL reaction volume, and the mixture contained 500 μg of hydrogenosomes (protein concentration) carrying both TvTom40-2-HA and Tom36-V5, import buffer (250 mM sucrose, 10 mM MOPS-KOH [pH 7.2], 3% BSA, 80 mM KCl, 7 mM MgCl_2_, and 10 mM ATP), 125 μL cytosolic extract, and 25 μL radiolabeled precursors at 37 °C. At each time point, 100 μL was removed and shifted to ice, and the hydrogenosomes were re-isolated and washed twice with import buffer. For the import-arrest experiment, the import assay was performed either in the presence or absence of 10 μM methotrexate (Sigma Aldrich) and 1 mM NADPH. Wherever indicated, the hydrogenosomes were treated with 50 μg/mL of proteinase K. For the import-arrest and coIP assay, the import assay was performed either in the presence or absence of 10 μM methotrexate, the hydrogenosomes obtained were subjected to crosslinking, and the HA-tagged protein was immunoprecipitated as described earlier except that 0.5% Triton X-100 was used to lyse the organelles instead of digitonin. The samples were electrophoresed, and the gel was vacuum dried. The gel was exposed for 4 to 5 days prior to phosphorimaging with Typhoon TLA 7000 scanner.

### Protein expression, pull-down, and binding assay

The gene encoding for the cytosolic domain of Tom36 and Tom46 (Tom36cd and Tom46cd) were cloned into pET42b vector tagged to polyhistidine at the C-terminus. The genes encoding for cytochrome b5 (TVAG_063210), frataxin (TVAG_182610), and αSCS (TVAG_165340; αSCS was fused to DHFR to the C-terminus) were subcloned into NEB PURExpress control plasmid, and the radiolabeled precursors were synthesised in the presence of L-[^35^S] methionine as described earlier. The recombinant His-tagged proteins were expressed in *E*. *coli* BL21 (DE3) strain at 37 °C for 3 hours following the induction with 0.5 mM IPTG. The cells from a 10 mL culture of *E*. *coli* (negative control) and strains expressing His-tagged proteins were harvested, resuspended in 4.5 mL lysis buffer (50 mM NaH_2_PO_4_, 300 mM NaCl, 10 mM imidazole, 1 mg/mL lysozyme, and EDTA-free cOmplete protease inhibitor cocktail), incubated on ice for 45 minutes, and lysed using QSonica sonicator. The homogenised extract was clarified at 9,000 rcf for 30 minutes at 4 °C. Aliquots of supernatant and pellet were used for immunoblotting to test the solubility of the proteins. The supernatant obtained was split into three equal parts and was incubated with 50 μL of Ni-NTA agarose resin (Qiagen) on an overhead rotator for 2 hours at room temperature. The resin collected was washed five times using 10 volumes of wash buffer (50 mM NaH_2_PO_4_, 300 mM NaCl, 20 mM imidazole, and EDTA-free cOmplete protease inhibitor cocktail). To block, the beads were washed thrice with wash buffer II (50 mM NaH_2_PO_4_, 300 mM NaCl, 20 mM imidazole, 3% BSA, and EDTA-free cOmplete protease inhibitor cocktail). To the mock-treated beads or beads bound with His-tagged protein, binding buffer (50 mM Tris, 150 mM NaCl [pH 7.4]), 50 μL of *Trichomonas* cytosolic extract, and 10 μL of radiolabeled precursors were added and incubated for 1 hour at 37 °C with gentle shaking. The beads were washed three times with the binding buffer, and the proteins were eluted with the elution buffer (50 mM NaH_2_PO_4_, 300 mM NaCl, 500 mM imidazole, and EDTA-free cOmplete protease inhibitor cocktail). The samples were electrophoresed, and the gel was vacuum dried. The gel was exposed for 4 to 5 days prior to phosphorimaging with Typhoon TLA 7000. The oligonucleotides used for cloning are listed in S4 Table.

### Antibody production

The gene encoding Sam50 was cloned into pET42b fused to a C-terminal His tag. The protein was expressed in *E*. *coli* BL21 (DE3) strain following an induction with 1 mM IPTG, and the His-tagged Sam50 was purified using affinity chromatography under denaturing conditions. The purified antigen was separated via SDS-PAGE, and the Coomassie-stained band was used to generate polyclonal antibody in rat.

## Supporting information

S1 Fig Comparison of the conserved β-motif of TvTom40-like proteins (TvTom40-1-7) with Tom40s and VDACs of other eukaryotes. The conserved residues of the β-motif, PxGxxHxH, are highlighted: P is polar (fluorescent green), x is any amino acid, G is glycine (fluorescent yellow), and H is hydrophobic (turquoise). All TvTom40 isoforms have the conserved β-motif except TvTom40-3, where the last hydrophobic residue has been replaced by serine. TOM, translocase of the outer membrane; TvTOM, *T*. *vaginalis* TOM; VDAC, voltage-dependent anion channel.
(PDF)

S2 Fig Conservation of TOM complex-forming residues. Highlighted residues mark anchoring positions for possible interactions between the Tom40 β-barrel and essential subunits of the TOM complex in *S*. *cerevisiae*. The selected 21 sequences were chosen out of the multiple alignment of 140 sequences to demonstrate the potential conservation of key residues and to highlight the differences between VDAC and Tom40 proteins. TOM, translocase of the outer membrane; TvTOM, *T*. *vaginalis* TOM; VDAC, voltage-dependent anion channel.
(PDF)

S3 Fig Sequence alignment of Tom22-like protein from *T*. *vaginalis* against Tom22 from other eukaryotes. Names of the organisms are as follows: *T*. *vaginalis*, *S*. *cerevisiae*, *Arabidopsis thaliana*, *Rattus rattus*, *Mus musculus*, *Homo sapiens*, and *Bos taurus*. The TMD is marked by a box, and the conserved residues are highlighted—tryptophan (yellow), hydroxylated residues (turquoise), and proline (green). TMD, transmembrane domain; Tom, translocase of the outer membrane.
(PDF)

S4 Fig Expression of His-tagged Tom36cd and Tom46cd in *E*. *coli* BL21 (DE3) strains. (A, B) Expression of Tom36cd-His and Tom46cd-His. SDS-PAGE gels stained with Coomassie and immunoblots probed with α-His antibody for the whole cell lysate from a 250 μL culture of *E*. *coli* strain expressing Tom36cd-His (panel A) and Tom46cd-His (panel B), respectively, before (Control) and 1 hour and 3 hours after induction with 0.5 mM IPTG. IPTG, Isopropyl β-D-1-thiogalactopyranoside; SDS-PAGE, sodium dodecyl sulphate-PAGE; Tom, translocase of the outer membrane.
(TIF)

S5 Fig EM analysis of the isolated TvTOM complex. (A, B) Preparation of purified TvTOM for EM analysis. (A) Immunoblot of digitonin-lysed extract of hydrogenosomes (Input; 5%) and the eluate (IP, 2.5%) from TvTom40-2-HA IP under native conditions using α-HA antibody. (B) Silver stained-gel showing the α-HA IP eluates from TvT1 WT strain and *Trichomonas* strain expressing HA-tagged TvTom40-2. Two bands marked were identified by MS as TvTom40-2. The common contaminant was identified as Cpn60. (C) Purified TvTOM complexes were applied on EM grids and negatively stained with phosphotungstic acid. Electron micrograph of negatively stained TvTOM complexes recorded at a magnification of 78,000×. Scale bar, 40 nm. Bottom panel: magnified view of selected particles with three, two, and one pore(s) (left to right). Scale bar, 10 nm. EM, electron microscopy; HA, human influenza hemagglutinin; In, Input; IP, immunoprecipitation; MS, mass spectrometry; TOM, translocase of the outer membrane; TvTOM, *T*. *vaginalis* TOM; WT, wild-type.
(PDF)

S6 Fig Enlarged version of the phylogenetic tree shown in Fig 11B. (PDF)

S1 Table HHpred search with each TvTom40 homologue against the NCBI conserved domains database (version 3.16) and *S*. *cerevisiae* proteome. NCBI, National Center for Biotechnology Information; TOM, translocase of the outer membrane; TvTom, *T*. *vaginalis* TOM.
(PDF)

S2 Table Pairwise comparison of HMM profiles for the seven TvTom40 homologues against PDB database using the HHpred tool. HMM, hidden Markov model; PDB, Protein Data Bank; TOM, translocase of the outer membrane; TvTOM, *T*. *vaginalis* TOM.
(PDF)

S3 Table TOM subunit orthologues identified in selected eukaryotic lineages. TOM, translocase of the outer membrane.
(XLSX)

S4 Table List of oligonucleotides. (PDF)

S1 Data A list of 24 well-annotated Tom40 sequences that were used to build Tom40 HMM. HMM, hidden Markov model; TOM, translocase of the outer membrane.
(TXT)

S2 Data A data set of proteins identified from TvTom40-2-HA, Tom36-HA, and Sam50-HA coIPs both under crosslinking and native conditions using LFQ-MS analysis. The data sets shown were obtained were four independent coIP experiments indicated by columns A, B, C, and D. A protein was considered enriched either if the protein was present only in the test sample and absent in the control or if the protein was enriched by a fold change of >1 in the test sample. Following are the column headings: accession number (protein ID on NCBI protein database or TrichDB), protein name, molecular weight of the protein, sequence coverage (percentage coverage of the peptide sequence to the full length protein sequence), peptides (number of peptides identified for a particular protein), unique peptides (number of unique peptides identified for a particular protein), score from the MS identification, intensity of the MS, MS/MS count. (A–D) Intensity from four independent IP experiments in binary logarithmic values; mean: arithmetic mean of intensity from four independent (A–D) IP experiments in binary logarithmic values; n: difference between mean of the test and the control samples; and fold change: actual change in the protein levels between the test and the control samples. coIP, co-immunoprecipitation; HA, human influenza hemagglutinin; LFQ-MS, label-free quantitative mass spectrometry; NCBI, National Center for Biotechnology Information; Sam, sorting and assembly machinery; TOM, translocase of the outer membrane; TrichDB, Trichomonas Genome Resource.
(XLSX)

S3 Data Protein sequences of the TOM subunit orthologues listed in S3 Table. TOM, translocase of the outer membrane.
(FASTA)

S4 Data A set of 1,114 proteins with their coordinates used for CLANS that were obtained from two iterations of PSI–BLAST with Tom36 and ATOM69 as queries. ATOM, archaic translocase of the outer membrane; CLANS, cluster analysis of sequences; TOM, translocase of the outer membrane.
(FASTA)

S5 Data An alignment of 418 TPR proteins from CLANS that were selected for the phylogenetic analysis. CLANS, cluster analysis of sequences; TPR, tetratricopeptide repeat.
(FASTA)

S6 Data A list of 447 Tom22 sequences that were used to build Tom22 HMM. HMM, hidden Markov model; Tom, translocase of the outer membrane.
(TXT)

S7 Data A list of 349 Tom7 sequences that were used to build Tom7 HMM. HMM, hidden Markov model; Tom, translocase of the outer membrane.
(TXT)

S8 Data Sequence alignments for TOM subunits that were used to identify orthologues in different eukaryotic lineages. Alignments of ATOM11, ATOM12, ATOM46, and ATOM69 homologues from kinetoplastids, Tom60 homologues from *Entamoeba* sp., and Tom36 homologues from parabasalids using MAFFT; Tom40 and VDAC (Porin_3) homologues, fungal Tom5, metazoan Tom5, plant Tom5, metazoan Tom6, fungal Tom6, Tom7, Tom20, plant Tom20, and Tom22 homologues from the Pfam database; plant Tom6 homologues from the Eggnog database; and Tom70 homologues from the COG database. ATOM, archaic TOM; COG, clusters of orthologous groups; MAFFT, multiple sequence alignment based on fast Fourier transform; Pfam, Protein families; Tom, translocase of the outer membrane.
(TXT)

S9 Data A list of Tom40 and VDAC sequences that were used for TvTom40-2 modelling. Tom, translocase of the outer membrane; TvTom, *T*. *vaginalis* TOM; VDAC, voltage-dependent anion channel.
(TXT)

## Abbreviations

αSCS: α-subunit of succinyl CoA synthetase
2D: two-dimensional
3D: three-dimensional
ATOM: archaic translocase of the outer membrane
BN-PAGE: blue native PAGE
CLANS: cluster analysis of sequences
CoA: coenzyme A
COG: clusters of orthologous groups
coIP: co-immunoprecipitation
Cryo-EM: Cryo electron microscopy
CTF: Contrast Transfer Function
cytME: cytoplasmic malic enzyme
DHFR: dihydrofolate reductase
DIC: differential interference contrast
Dox: doxycycline
ERAD: endoplasmic reticulum-associated protein degradation
FASP: filter-aided sample preparation
Fdx: ferredoxin
Fis1: mitochondrial fission 1
HA: human influenza hemagglutinin
HMM: hidden Markov model
Hmp: hydrogenosomal membrane protein
Homp: hydrogenosomal outer membrane protein
Hsp: heat shock protein
IMS: intermembrane space
IPTG: Isopropyl β-D-1-thiogalactopyranoside
ITS: internal-targeting sequence
LECA: last common eukaryotic ancestor
LFQ-MS: label-free quantitative mass spectrometry
MAFFT: multiple sequence alignment based on fast Fourier transform
MS: mass spectrometry
NCBI: National Center for Biotechnology Information
Ni-NTA: Ni-nitrilotriacetic acid
NTS: N-terminal targeting sequence
OD: optical density
OMM: outer mitochondrial membrane
PDB: Protein Data Bank
Pfam: Protein families
SAM: sorting and assembly machinery
SAR: Stramenopiles, Alveolata and Rhizaria
SD-Leu: synthetic drop-out medium without leucine
SDS-PAGE: sodium dodecyl sulphate-PAGE
STED: Stimulated Emission Depletion
TA: tail-anchored
TCA: tricarboxylic acid
TEM: transmission electron microscopy
TIM: translocase of the inner membrane
TMD: transmembrane domain
TMHMM: transmembrane helices HMM
TOM: translocase of the outer membrane
TPR: tetratricopeptide repeat
TrichDB: Trichomonas Genome Resource
TvTOM: *T*. *vaginalis* TOM
VDAC: voltage-dependent anion channel
YPG: yeast extract-peptone-glycerol
