# Supplementary material for: Triplet-pore structure of a highly divergent TOM complex of hydrogenosomes in Trichomonas vaginalis
Source: PLoS Biol. 2019 Jan 4;17(1):e3000098. doi: 10.1371/journal.pbio.3000098 (PMC6334971; doi:10.1371/journal.pbio.3000098)
Supplement: S1 Fig — The conserved residues of the β-motif, PxGxxHxH, are highlighted: P is polar (fluorescent green), x is any amino acid, G is glycine (fluorescent yellow), and H is hydrophobic (turquoise). All TvTom40 isoforms have the conserved β-motif except TvTom40-3, where the last hydrophobic residue has been replaced by serine. TOM, translocase of the outer membrane; TvTOM, T. vaginalis TOM; VDAC, voltage-dependent anion channel. (PDF) [file pbio.3000098.s001.pdf]

```
                               PxGxxHxH

TvTom40-1      ICGTLNHKAKSYTIGLGFVFNPDDFVST--------------------      308
TvTom40-2      VSTHLDHKNAQYNFGLGFQWVENSTN----------------------      290
TvTom40-3      VASILDHPAKNYKLGLGFYSQ---------------------------      305
TvTom40-4      VNCCLNHLEADYSFGIDLSINQ--------------------------      296
TvTom40-5      ISSFADHFQKLYSLGMAVSVRDTSSD----------------------      297
TvTom40-6      LTSQADIFRKHYNFGLGLVFA---------------------------      298
TvTom40-7      VGLNMLKQINAISLGLQLDTGKQI------------------------      293
SpsTom40       EVGGKFKLGGAPEVGFSLVV----------------------------      275
GlTom40        VIGSLDIFSGRTTVGVGLVLSEQALPRFIHKAVRKANSSSNHK-----      355
NbTom40        VNMNCEIGKGEFIYGYGLNYYF--------------------------      280
NcTom40        FAADVDHVTQQAKLGMSVSIEASDVDL-QEQQEGAQSLNIPF------      349
SpTom40        FSSELDHPNRNAKVGLGLSLELPGSDEMIQQQQQQLAAQTA-------      344
ScTom40        FCGEIDHFKNDTKIGCGLQFETAGNQELLMLQQGLDADGNPLQALPQL      387
EsTom40        ISSSLDHSEDKQTFGLALTCGQ--------------------------      442
DmTom40        LSGRMNHVKNNFRLGCGLMIG---------------------------      344
RnTom40        LCAFLNHRKNKFLCGFGLTIG---------------------------      361
MmTom40        LCAFLNHRKNKFLCGFGLTIG---------------------------      361
SlsTom40       LGSFLNHKKNKFQCGFGITIG---------------------------      334
TpTom40        LSGDLNYLANEYKFGLGLILGQ--------------------------      280
AtTom40        LSAELDHKKKDYKFGFGLTVG---------------------------      309
CrTom40        LSAEMDHWQSNYKFGFGIVAGE--------------------------      339
TaVDAC         AEVDTKAIEKSSKVGIAIALKP--------------------------      275
BrVDAC         GEVDSKAIEKSAKVGFALALKP--------------------------      276
StVDAC         GEVDTRAIEKSAKIGLAVALKP--------------------------      276
McVDAC         GEVDTKAIEKSAKVGLALALKP--------------------------      276
CrVDAC         LLNGKNLNEGGNKLSLGLELEA--------------------------      283
HsVDAC         LVDGKSINAGGHKVGLALELEA--------------------------      294
SaVDAC         LIDGKNFHAGGHKVGMGFELEA--------------------------      283
SoVDAC         LIDGKNFNQGGHKIGLALELEA--------------------------      282
LmVDAC         LIDGKNFNQGGHKIGVALEMEA--------------------------      282
DmVDAC         LVDGKNFNAGGHKIGVGLELEA--------------------------      282
AaVDAC         MIDGKSFNTGGHKIGVALELEA--------------------------      282
AgVDAC         LVDGKNFNAGGHKIGVALELEA--------------------------      282
```
