# Supplementary material for: Triplet-pore structure of a highly divergent TOM complex of hydrogenosomes in Trichomonas vaginalis
Source: PLoS Biol. 2019 Jan 4;17(1):e3000098. doi: 10.1371/journal.pbio.3000098 (PMC6334971; doi:10.1371/journal.pbio.3000098)
Supplement: S2 Fig — Highlighted residues mark anchoring positions for possible interactions between the Tom40 β-barrel and essential subunits of the TOM complex in S. cerevisiae. The selected 21 sequences were chosen out of the multiple alignment of 140 sequences to demonstrate the potential conservation of key residues and to highlight the differences between VDAC and Tom40 proteins. TOM, translocase of the outer membrane; TvTOM, T. vaginalis TOM; VDAC, voltage-dependent anion channel. (PDF) [file pbio.3000098.s002.pdf]

```
                                   80        90       100       110       120       130       140
                          ....|....|....|....|....|....|....|....|....|....|....|....|....|....|
sp|O96008|TOM40_HUMA      SERTPGAATASASGAAEDGACGCLPN---PGT---------FEECHRKC-KELFP---IQMEGVKLT-VN 106
sp|Q9QYA2|TOM40_MOUS      SERTPGAAASGAAAASEDGSCGCLPN---PGT---------FEECHRKC-KELFP---VQMEGVKLT-VN 106
sp|Q7ZTM6|TOM40_XENL      ------------TPDKKEPQEERLPN---PGT---------FEECHRKC-KELFP---IQMEGVKLI-VN 81
sp|Q9U4L6|TO401_DROM      --------------SLAAAKDAALEN---PGT---------VEELHKKC-KDIQA---ITFEGAKIM-LN 86
sp|Q18090|TOM40_CAEE      --------------ESELASPIPQTN---PGS---------YEELHRKA-RDVFP---TCFEGAKLM-VN 44
sp|P23644|TOM40_YEAS      --------------LHSHRQSLELVN---PGT---------VENLNKEVSRDVFLSQ-YFFTGLRAD-LN 89
sp|P24391|TOM40_NEUC      --------------FQERRKQFGLSN---PGT---------IETIAREVQRDTLLTN-YMFSGLRAD-VT 70
sp|P21796|VDAC1_HUMA      -----------------------MAV---PPT---------YADLGKSA-RDVFTKG-YGFGLIKLD-LK 32
sp|Q60932|VDAC1_MOUS      -----------------LLLWQNMAV---PPT---------YADLGKSA-RDVFTKG-YGFGLIKLD-LK 45
tr|Q7ZWZ0|VDAC_XENLA      -----------------------MAI---PPA---------YVDLGKSA-RDIFTKG-YGFGFIKLD-LK 32
sp|Q94920|VDAC_DROME      ------------------------MA---PPS---------YSDLGKQA-RDIFSKG-YNFGLWKLD-LK 31
sp|Q21752|VDAC_CAEEL      ------------------------MA---PPT---------FADLGKSA-KDLFNKG-YNFGFLKID-ST 31
sp|P04840|VDAC1_YEAS      ------------------------MS---PPV---------YSDISRNI-NDLLNKDFYHATPAAFD-VQ 32
sp|P07144|VDAC_NEUCR      ------------------------MA---VPA---------FSDIAKSA-NDLLNKDFYHLAAGTIE-VK 32
TVAG_399510_Tom40-1       ----------------------ILES---------------FSQVTSRD-NAILEL--PYYPGLKLE-AS 30
TVAG_332970_Tom40-2       -----------------------------SGTEY-------YESM-----NNLFDN--KNFPGIKFD-VE 28
TVAG_450220_Tom40-3       ----------------------MLSS---------------FLHTNLRH-QEIFDV--GAFSGLNITFDS 30
TVAG_123100_Tom40-4       -----------------------------PKTEQTVTLLPQYEDINAEN-DVMFTL--NDTKGLSLK-LG 42
TVAG_341190_Tom40-5       ----------------------MIFD---PKQ----TKCPEFEAFESEI-VGPTSL--PPNNTFEIV-VA 37
TVAG_195900_Tom40-6       ----------------------STPEQKAPEL------VGDFDTLLGKV-TDVASL--NLINGFEFT-HV 43
TVAG_044000_Tom40-7       ----------------------TVPS---------------YQTIGSAL-QNVCYT--GEQPGLSFL-HT 34

                                  150       160       170       180       190       200       210
                          ....|....|....|....|....|....|....|....|....|....|....|....|....|....|
sp|O96008|TOM40_HUMA      KGLS-------------NHFQVNHTVALSTI-----GE-----------SNYHFGVTY--VGTKQLSPTE 145
sp|Q9QYA2|TOM40_MOUS      KGLS-------------NRFQVTHTVALGTI-----GE-----------SNYHFGVTY--VGTKQLSPTE 145
sp|Q7ZTM6|TOM40_XENL      KGLS-------------NYFQVNHTISLSTI-----GE-----------SNYHFGATY--VGTKQLGPAE 120
sp|Q9U4L6|TO401_DROM      KGLS-------------NHFQVSHTINMSNV-----VP-----------SGYRFGATY--VGTKEFSPTE 125
sp|Q18090|TOM40_CAEE      KGLS-------------SHFQVSHTLSLSAM---N--------------TGYRFGATY--VGTNQVGPAE 82
sp|P23644|TOM40_YEAS      KAFSMN-----------PAFQTSHTFSIGSQ---A-L------------PKYAFSALF--ANDN------ 124
sp|P24391|TOM40_NEUC      KAFSLA-----------PLFQVSHQFAMGER---L--------------NPYAFAALY--GTNQ------ 104
sp|P21796|VDAC1_HUMA      TKSENG-----------LEFTSSGSANTETT---K--------------VTGSLETKY--RWTEY----- 67
sp|Q60932|VDAC1_MOUS      TKSENG-----------LEFTSSGSANTETT---K--------------VNGSLETKY--RWTEY----- 80
tr|Q7ZWZ0|VDAC_XENLA      TKSENG-----------LEFTSSGSANAETS---K--------------VSGNLETKY--KWAEY----- 67
sp|Q94920|VDAC_DROME      TKTSSG-----------IEFNTAGHSNQESG---K--------------VFGSLETKY--KVKDY----- 66
sp|Q21752|VDAC_CAEEL      TRAGDNKE---------VEFKSAASHNIGSG---K--------------LGGNLDVKY--KIPQY----- 68
sp|P04840|VDAC1_YEAS      TTTANG-----------IKFSLKAKQPVKDG---P--------------LSTNVEAKL--NDKQT----- 67
sp|P07144|VDAC_NEUCR      SNTPNN-----------VAFKVTG-KSTHDK---V--------------TSGALEGKF--TDKPN----- 66
TVAG_399510_Tom40-1       YNLGNDAYFHSYVSFRPLKISIVPGPNGQYN---S--------------IQNQTAVAY--TTFK------ 75
TVAG_332970_Tom40-2       LNQS-------------PETTINSSINIRHPYFNI--------------IEKNSQTKI--VPS------- 62
TVAG_450220_Tom40-3       PTQSKDTTISTVIFLQPLSISIVRGEGGKPE---T--------------RSNQTTNVK--FNYQ------ 75
TVAG_123100_Tom40-4       GKGKFD-----------VNYSIGGALRPTKV---AIVP----Q------VNGEFSILSLPIRS------- 81
TVAG_341190_Tom40-5       KKLK-------------DIGNLISVLSIKPR---L-YPLKYADQAGEQRLPGNAAVNF--TTDKI----- 83
TVAG_195900_Tom40-6       YQPQ-------------PSHSIVQTISISPR---I-HPVELAAQAGEKVIPGNYSAVF--VGT------- 87
TVAG_044000_Tom40-7       HSVD-------------MSTNFTQILNIGPR---I-YPLALIDQSHEEIFPGSYSAIL--KTE------- 78

                                  430       440       450       460       470       480       490
                          ....|....|....|....|....|....|....|....|....|....|....|....|....|....|
sp|O96008|TOM40_HUMA      TRM--QDTSVSFGYQLDLPKANLLFKGSVDSNWIVGATLEKKL-PPLPLTLALGAFLNHRKNKF---QCG 355
sp|Q9QYA2|TOM40_MOUS      TRM--QDTSASFGYQLDLPKANFLFKGSVNSNWIVGATLEKKL-PPLPLTLSLCAFLNHRKNKF---LCG 355
sp|Q7ZTM6|TOM40_XENL      ARM--QDTSVSLGYQLDLPKANLLFKGSIDSNWIVGATLEKKL-PPLPLTLAMGAFLNHKKNKF---QCG 330
sp|Q9U4L6|TO401_DROM      LRM--QESVATLAYQIDLPKANLVFRGGIDSNWQIFGVLEKRL-APLPFTLALSGRMNHVKNNF---RLG 338
sp|Q18090|TOM40_CAEE      ANV--GEAVTTLAYQTELPEEGVTMRASFDTNWTVGGVFEKRLSQQLPFTLALSGTLNHVKAAG---KFG 295
sp|P23644|TOM40_YEAS      QPT--VEGSTTIGAKYEYRQS--VYRGTLDSNGKVACFLERKV-LP-TLSVLFCGEIDHFKNDT---KIG 354
sp|P24391|TOM40_NEUC      GLT--KEGITTFGAKYDFRMS--TFRAQIDSKGKLSCLLEKRLGAA-PVTLTFAADVDHVTQQA---KLG 323
sp|P21796|VDAC1_HUMA      AGN--SNTRFGIAAKYQIDPDA-CFSAKVNNSSLIGLGYTQTL-KP-GIKLTLSALLDGKNVNAGGHKLG 276
sp|Q60932|VDAC1_MOUS      AGN--SNTRFGIAAKYQVDPDA-CFSAKVNNSSLIGLGYTQTL-KP-GIKLTLSALLDGKNVNAGGHKLG 289
tr|Q7ZWZ0|VDAC_XENLA      AGN--SNTRFGIAAKYQIDSDA-SFSAKVNNSSLIGLGYTQTL-KP-GIKLTLSTLVDGKNINAGGHKLG 276
sp|Q94920|VDAC_DROME      SGT--SNTKFAIGAKYQLDDDA-SVRAKVNNASQVGLGYQQKL-RD-GVTLTLSTLVDGKNFNAGGHKIG 275
sp|Q21752|VDAC_CAEEL      VGG--NGADYALATKYAPSRDL-TVRAKVNSSSQVAVAATHSL-SP-ALKLTLSTQFNLAANDA--HKFG 274
sp|P04840|VDAC1_YEAS      CKLPNSNVNIEFATRYLPDASS-QVKAKVSDSGIVTLAYKQLL-RP-GVTLGVGSSFDALKLSEPVHKLG 276
sp|P07144|VDAC_NEUCR      SKT-GNTVGLEVATKYRIDPVS-FVKGKINDRGVAAIAYNVLL-RE-GVTLGVGASFDTQKLDQATHKVG 276
TVAG_399510_Tom40-1       STA--LLPYLTIASTVELGRS--KVQTNV-SDKLISSLYSYKV-SD-NMNFEICGTLNHKAKSY---TIG 295
TVAG_332970_Tom40-2       -SN--LNSKVELAWIAKVNDA--IIHSSIDTKLNVSTVYKQEL-YP-QCNILVSTHLDHKNAQY---NFG 279
TVAG_450220_Tom40-3       SKY--IEPRLQLTCKANLGKS--TVHSSILTTGVVESKFTSKV-SD-TFTITVASILDHPAKNY---KLG 299
TVAG_123100_Tom40-4       RMP--AFVAADLSWIYTNKDT--QVHSIISTNGDVFSELSRKL-NE-KVALNVNCCLNHLEADY---SFG 289
TVAG_341190_Tom40-5       PST--LQTDILFGFQRAFMSS--KVSAVMNTKGSVGSVFQKDV-NE-KYSIIISSFADHFQKLY---SLG 286
TVAG_195900_Tom40-6       PSE--LRAKSAFFINRNFAMT--VFAFNVTSDATISSSLQRRF-GK-GYQMTLTSQADIFRKHY---NFG 292
TVAG_044000_Tom40-7       HDA--NIKVGTLGYNMVIGNN--SVCASISSELSTCAKFVRDT-GK-GLTVSVGLTSNMLKQIN---AIS 282
```
