# Supplementary material for: Triplet-pore structure of a highly divergent TOM complex of hydrogenosomes in Trichomonas vaginalis
Source: PLoS Biol. 2019 Jan 4;17(1):e3000098. doi: 10.1371/journal.pbio.3000098 (PMC6334971; doi:10.1371/journal.pbio.3000098)
Supplement: S3 Fig — Names of the organisms are as follows: T. vaginalis, S. cerevisiae, Arabidopsis thaliana, Rattus rattus, Mus musculus, Homo sapiens, and Bos taurus. The TMD is marked by a box, and the conserved residues are highlighted—tryptophan (yellow), hydroxylated residues (turquoise), and proline (green). TMD, transmembrane domain; Tom, translocase of the outer membrane. (PDF) [file pbio.3000098.s003.pdf]

```
Tom22-like     ------------------------------------------------------------
ScTom22        MVELTEIKDDVVQLDEPQFSRNQAIVEEKASATNNDVVDDEDDSDSDFEDEFDENETLLD
AtTom22        ------------------------------------------------------------
RatTom22       MAAAVA----AAGAGEPL-SPEELVPKAEAEKAEEDL-------EEDDDD--ELDETLSE
MusTom22       MAAAVA----AAGAGEPL-SPEELLPKAEAEKAEEEL-------EEDDDD--ELDETLSE
HsTom22        MAAAVA----AAGAGEPQ-SPDELLPKGDAEKPEEEL-------EEDDDE--ELDETLSE
BtTom22        --MAAA----AAGPGAPL-SADELLPKGDAEKPEEEL-------EEEDDE--ELDETLSE


Tom22-like     ---------------------------MFNLVSRKK-FYKKKMMMMPPPQPSKLEQITQI
ScTom22        RIVALKDIVPPGKRQTIS------------------NFFGFT--------SSFVRNAFTK
AtTom22        -------MAPKKIGAGKGDSSILAKISNYDIVSQGRRAACDA--------VYVSKKLLKS
RatTom22       RLWGLTEMFPERVRSAAG------------------ATFDLS--------LFVAQKMYRF
MusTom22       RLWGLTEMFPERVRSAAG------------------ATFDLS--------LFVAQKMYRF
HsTom22        RLWGLTEMFPERVRSAAG------------------ATFDLS--------LFVAQKMYRF
BtTom22        RLWGLTEMFPERVRSAAG------------------ATFDLS--------LFVAQKMYRF
                                                                      .:

Tom22-like     VKETAWVIGTSAFVIIFPIYVATKL-----------------------------------
ScTom22        SGNIAWTLTTTALLLGVPLSLSILAEQQLIEMEKTFDLQSDANNILAQGEKDAAATAN--
AtTom22        TGKAAWIAGTTFLILAVPLILELEQDHRLGEI----DFEQ--ASLLGTPPVGAML-----
RatTom22       SRAALWIGTTSFMILVLPVVFETEKLQMEQQQ----QLQQ--RQILLGPNTGLSGGMPGA
MusTom22       SRAALWIGTTSFMILVLPVVFETEKLQMEQQQ----QLQQ--RQILLGPNTGLSGGMPGA
HsTom22        SRAALWIGTTSFMILVLPVVFETEKLQMEQQQ----QLQQ--RQILLGPNTGLSGGMPGA
BtTom22        SRAALWIGTTSFMILVLPVVFETEKLQMEQQQ----QLQQ--RQILLGPNTGLSGGMPGA
                    *   *: ::: .*: .

Tom22-like     --------
ScTom22        --------
AtTom22        --------
RatTom22       LPPLPGKI
MusTom22       LPPLPGKM
HsTom22        LPSLPGKI
BtTom22        LPSLPGKI
```
