# Supplementary figures and images for: Triplet-pore structure of a highly divergent TOM complex of hydrogenosomes in Trichomonas vaginalis
Source: PLoS Biol. 2019 Jan 4;17(1):e3000098. doi: 10.1371/journal.pbio.3000098 (PMC6334971; doi:10.1371/journal.pbio.3000098)

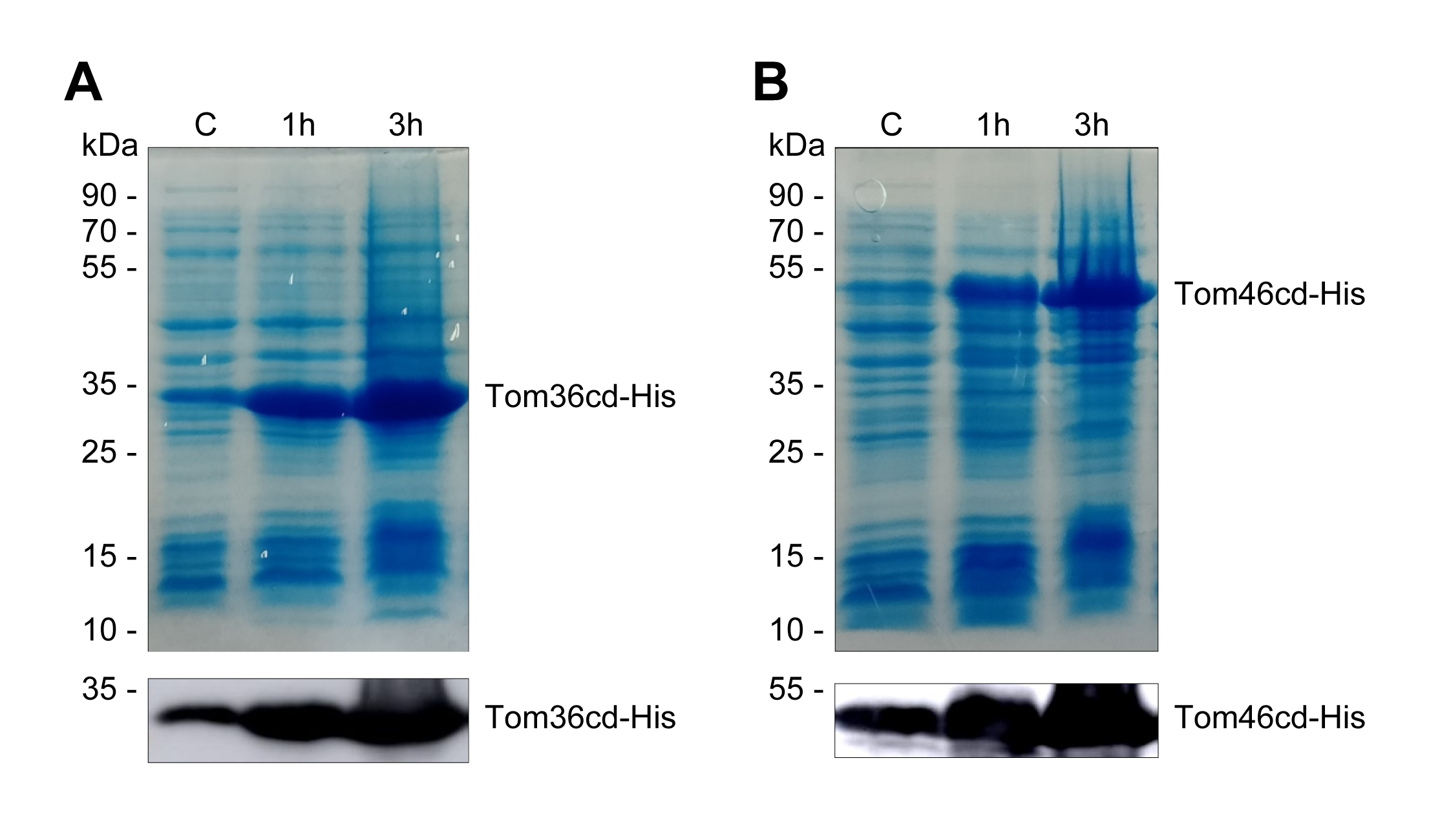

Supplement: S4 Fig — (A, B) Expression of Tom36cd-His and Tom46cd-His. SDS-PAGE gels stained with Coomassie and immunoblots probed with α-His antibody for the whole cell lysate from a 250 μL culture of E. coli strain expressing Tom36cd-His (panel A) and Tom46cd-His (panel B), respectively, before (Control) and 1 hour and 3 hours after induction with 0.5 mM IPTG. IPTG, Isopropyl β-D-1-thiogalactopyranoside; SDS-PAGE, sodium dodecyl sulphate-PAGE; Tom, translocase of the outer membrane. (TIF) [file pbio.3000098.s004.tif]

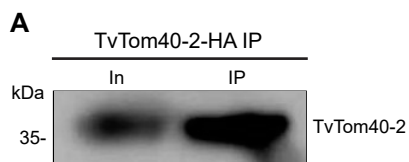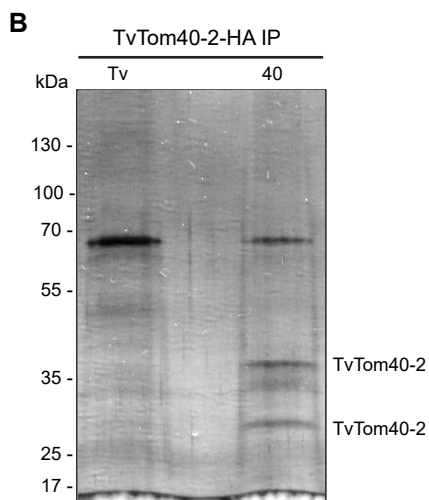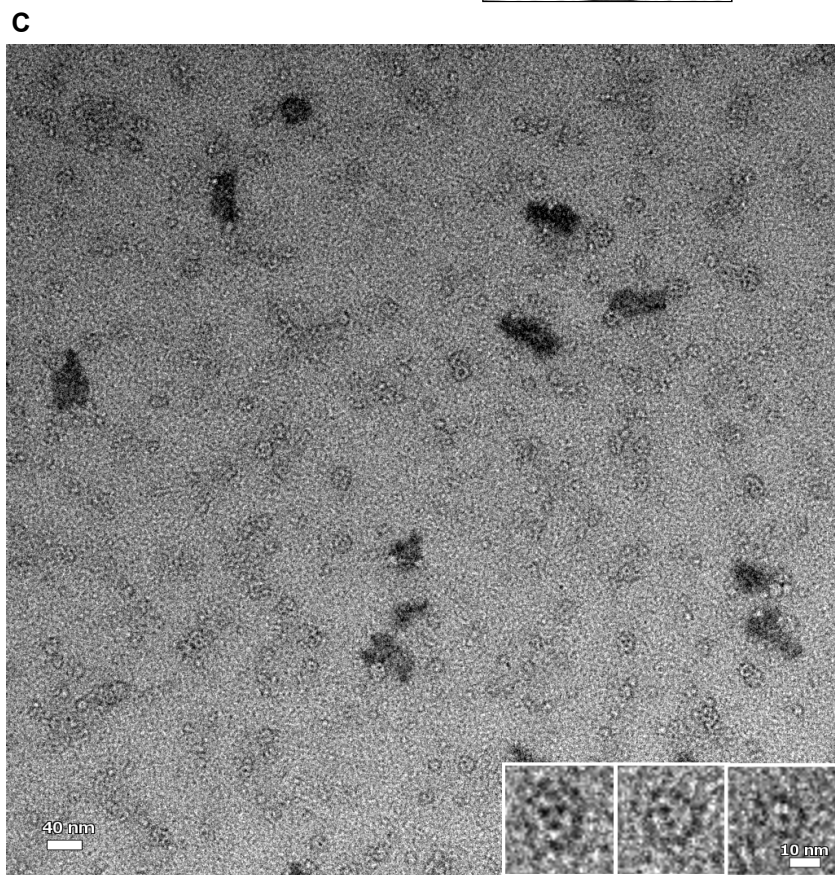

Supplement: S5 Fig — (A, B) Preparation of purified TvTOM for EM analysis. (A) Immunoblot of digitonin-lysed extract of hydrogenosomes (Input; 5%) and the eluate (IP, 2.5%) from TvTom40-2-HA IP under native conditions using α-HA antibody. (B) Silver stained-gel showing the α-HA IP eluates from TvT1 WT strain and Trichomonas strain expressing HA-tagged TvTom40-2. Two bands marked were identified by MS as TvTom40-2. The common contaminant was identified as Cpn60. (C) Purified TvTOM complexes were applied on EM grids and negatively stained with phosphotungstic acid. Electron micrograph of negatively stained TvTOM complexes recorded at a magnification of 78,000×. Scale bar, 40 nm. Bottom panel: magnified view of selected particles with three, two, and one pore(s) (left to right). Scale bar, 10 nm. EM, electron microscopy; HA, human influenza hemagglutinin; In, Input; IP, immunoprecipitation; MS, mass spectrometry; TOM, translocase of the outer membrane; TvTOM, T. vaginalis TOM; WT, wild-type. (PDF) [file pbio.3000098.s005.pdf]

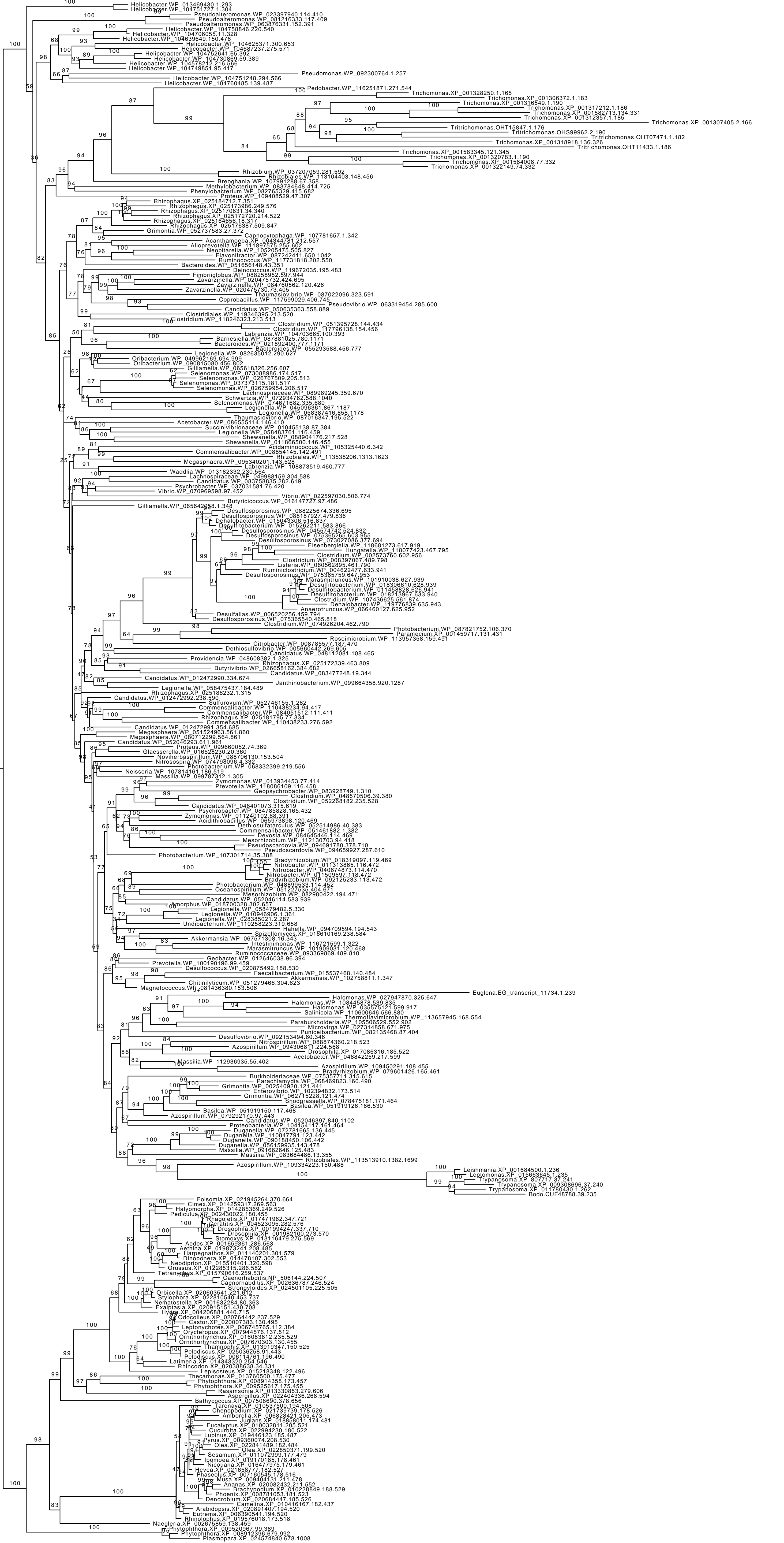

0.8

Supplement: S6 Fig — (PDF) [file pbio.3000098.s006.pdf]
