# Supplementary material for: Triplet-pore structure of a highly divergent TOM complex of hydrogenosomes in Trichomonas vaginalis
Source: PLoS Biol. 2019 Jan 4;17(1):e3000098. doi: 10.1371/journal.pbio.3000098 (PMC6334971; doi:10.1371/journal.pbio.3000098)
Supplement: S1 Table — NCBI, National Center for Biotechnology Information; TOM, translocase of the outer membrane; TvTom, T. vaginalis TOM. (PDF) [file pbio.3000098.s007.pdf]

TvTom40 homologue	Hit	Name	E-value
TvTom40-1	NP_012152.3	putative porin POR2 [*Saccharomyces cerevisiae* S288c]	2.80E-34
TvTom40-2	NP_013930.1	Tom40p [*Saccharomyces cerevisiae* S288c]	4.30E-42
TvTom40-3	NP_013930.1	Tom40p [*Saccharomyces cerevisiae* S288c]	1.00E-33
TvTom40-4	NP_012152.3	putative porin POR2 [*Saccharomyces cerevisiae* S288c]	0.0000096
TvTom40-5	NP_013930.1	Tom40p [*Saccharomyces cerevisiae* S288c]	2.10E-30
TvTom40-6	cd07305	Porin3_Tom40; Translocase of outer mitochondrial membrane 40 (Tom40). Tom40 forms a channel in the mitochondrial outer membrane with a pore about 1.	0.0000018
TvTom40-7	cd07305	Porin3_Tom40; Translocase of outer mitochondrial membrane 40 (Tom40). Tom40 forms a channel in the mitochondrial outer membrane with a pore about 1.	0.0000012
