# Supplementary material for: Triplet-pore structure of a highly divergent TOM complex of hydrogenosomes in Trichomonas vaginalis
Source: PLoS Biol. 2019 Jan 4;17(1):e3000098. doi: 10.1371/journal.pbio.3000098 (PMC6334971; doi:10.1371/journal.pbio.3000098)
Supplement: S2 Table — HMM, hidden Markov model; PDB, Protein Data Bank; TOM, translocase of the outer membrane; TvTOM, T. vaginalis TOM. (PDF) [file pbio.3000098.s008.pdf]

**Tom40 homologue**	**Hit**	**Family**	**E-value**
TvTom40-1	5O8O_A	TOM40	3.80E-29
	5JDP_A	VDAC	1.20E-36
TvTom40-2	5O8O_A	TOM40	4.30E-38
	4C69_X	VDAC	1.60E-35
TvTom40-3	5O8O_A	TOM40	3.80E-31
	4C69_X	VDAC	3.20E-30
TvTom40-4	5O8O_A	TOM40	1.30E-03
	4C69_X	VDAC	4.90E-05
TvTom40-5	5O8O_A	TOM40	2.60E-26
	4C69_X	VDAC	1.10E-23
TvTom40-6	5O8O_A	TOM40	3.40E-05
	4C69_X	VDAC	4.60E-04
TvTom40-7	5O8O_A	TOM40	4.30E-05
	4C69_X	VDAC	1.00E-04
