# Supplementary material for: Triplet-pore structure of a highly divergent TOM complex of hydrogenosomes in Trichomonas vaginalis
Source: PLoS Biol. 2019 Jan 4;17(1):e3000098. doi: 10.1371/journal.pbio.3000098 (PMC6334971; doi:10.1371/journal.pbio.3000098)
Supplement: S4 Table — (PDF) [file pbio.3000098.s010.pdf]

Primer name	Sequence: 5'- 3'
TvTom40-2-NdeI-F	GGCCCATATGAAATCTGGAACTGAATATTATG
TvTom40-2-BamHI-R	GATAGGATCCATTAGTGGAGTTTTCAAC
TvTom40-2-EcoRI-F	GTCTCGGAATTCATGAAATCTGGAACTGAATATTATG
TvTom40-2-SmaI-R	GTCTCGCCCGGGATTAGTGGAGTTTTCAACCC
Tom46-NdeI-F	GGCCCATATGGAAGTTGATTTAGTAATTCC
Tom46-BglII-R	GGACAGATCTTTTCTTCATGAGTTTCTTTGC
Homp38-NdeI-F	GGCCCATATGATTACTCCATCTCATC
Homp38-BglII-R	AACCAGATCTTTTTCTTGAGAATTTCTTGTAAATTGC
Tom22-like-NdeI-F	GGCCCATATGTTCAACTTGGTCTCAAG
Tom22-like-BamHI-R	TATAGGATCCGAGCTTCGTTGCGACG
TvFdx1-NdeI-F	AATTCATATGCTCTCTCAAGTTTGCCGC
TvFdx1-NdeI-R	AATTCATATGGAGCTCGAAAACAGCACCATCG
TJ264	CTTTGAAAGCGGTGTATGCGTATAAGGCCTTTTAACCAT<br>CGTGCAAAACACAGTATAGCGACCAGCATTCACATACG
TJ265	GGGATAGTGGGAATTTGAGAGGCCTCTGCTAATGGAGTT<br>GGTGCAGACATAAGCTTATCGATACCGTCGATCCCC
Tom36cd-NdeI-F	TGACCATATGGAGAACGCATTTTTGATGACTCC
Tom36cd-XhoI-R	TGAGCTCGAGAACTTTAGAAAGATTGAATGGGATTCC
Tom46cd-XhoI-R	TGAGCTCGAGTGCTTTTGTATTAATTGTCGGATATTC
